# Supplementary material for: Near Neutral Selectionist Theories (NNST) for SARS-CoV-2 suggested by the substitution-mutation ratio (c/µ) analysis
Source: PLoS One. 2026 Mar 4;21(3):e0343410. doi: 10.1371/journal.pone.0343410 (PMC12959723; doi:10.1371/journal.pone.0343410)
Supplement: S1 Table — Reported nucleotide mutations in the cell-based SARS-CoV-2 studies analyzed in our previous study, their locations within the SARS-CoV-2 genome, and the percent substitution rate at these sites computed from our SARS-CoV-2 genomic sequence datasets. (PDF) [file pone.0343410.s001.pdf]

Table S1. Nucleotide point mutation data from cell infection experiments. Reported nucleotide mutations in the cell-based SARS-COV-2 studies analyzed in our previous study, their locations within the SARS-CoV-2 genome, and the percent substitution rate at these sites computed from our SARS-CoV-2 genomic sequence datasets.

Gene	Nucleotide Mutation Reported	%Substitution Rate	Reference
NSP5	G10870T	0.13	Bamford
NSP11	C14318T	0.04	Bamford
S	C23997G	0.05	Bamford
Orf8	C28253T	3.50	Bamford
NSP4	C8782T	1.03	Gamage
S	C23525T	2.31	Gamage
S	T24013A	0.01	Gamage
S	A25381X	0.00	Gamage
Orf3a	C25568A	0.00	Gamage
M	G27147C	0.07	Gamage
S	A25381C	0.00	Meganck
NSP14	T19605C	0.00	Ogando
S	T22303G	0.00	Ogando
Orf3a	G26144T	0.05	Ogando
NSP2	C2388T	0.08	Araujo
S	T21784A	0.00	Araujo
NSP3	C3037T	37.06	Pohl
NSP6	A11728G	0.00	Pohl
S	T22303G	0.00	Pohl
S	A23403G	37.47	Pohl
S	C23606T	0.00	Pohl
NSP2	A1515G	0.01	Pohl
NSP2	A2550C	0.03	Pohl
NSP4	C9223T	0.04	Pohl
NSP6	C11074T	0.11	Pohl
NSP6	G11083T	1.79	Pohl
NSP11	C14805T	0.93	Pohl
NSP12	T17247C	0.03	Pohl
S	G22205A	0.12	Pohl
Orf3a	G26144T	0.25	Pohl
Orf6	A27241G	0.00	Pohl
S	G22343T	0.00	Feng
S	A23063T	13.06	Feng
S	C23271A	9.15	Feng
S	A23403G	37.47	Feng
S	C23604A	18.58	Feng
S	C23709T	9.20	Feng
S	T24236G	9.12	Feng
S	G24914C	9.37	Feng
NSP13	G18286T	0.00	Chen
S	T21784A	0.00	Chen
NSP2	C2206A	0.00	Chen
E	C26261T	0.00	Chen
Orf6	T27348G	0.00	Chen
N	A28996C	0.00	Chen
NSP2	C1567T	0.23	Chen
S	T21784G	0.00	Chen
S	G23285T	0.01	Chen

S	T24754C	0.00	Chen
E	C26270T	0.03	Savellini
M	A26709T	0.01	Savellini
N	C28310T	0.32	Savellini
N	G28880A	0.01	Savellini
N	G28883A	19.06	Savellini
NSP1	C560T	0.01	Savellini
NSP3	A2831G	0.00	Savellini
NSP3	G6512T	0.00	Savellini
NSP3	G8393A	0.00	Savellini
NSP4	C10028T	0.00	Savellini
NSP5	C10448A	0.01	Savellini
NSP6	A11537G	0.02	Savellini
NSP6	C11393T	0.00	Touret
NSP6	T11687C	0.01	Touret

***%Substitution Rate (c) = (#nucleotide substitutions / nucleotide site * 19 months) * 100%.**

References

Bamford CGG, Broadbent L, Aranday-Cortes E, McCabe M, McKenna J, Courtney DG, Touzelet O, Ali A, Roberts G, Campos GL, Simpson D, McCaughey C, Fairley D, Mills K, Power UF, Breathing Together I (2022) Comparison of SARS-CoV-2 Evolution in Paediatric Primary Airway Epithelial Cell Cultures Compared with Vero-Derived Cell Lines. Viruses-Basel 14:1

Gamage AM, Tan KS, Chan WOY, Liu J, Tan CW, Ong YK, Thong M, Andiappan AK, Anderson DE, Wang D, Wang LF (2020) Infection of human Nasal Epithelial Cells with SARS-CoV-2 and a 382-nt deletion isolate lacking ORF8 reveals similar viral kinetics and host transcriptional profiles. Plos Pathogens 16:1

Meganck RM, Edwards CE, Mallory ML, Lee RE, Dang H, Bailey AB, Wykoff JA, Gallant SC, Zhu DR, Yount BL, Kato T, Shaffer KM, Nakano S, Cawley AM, Sontake V, Wang JR, Hagan RS, Miller MB, Tata PR, Randell SH, Tse LV, Ehre C, Okuda K, Boucher RC, Baric RS (2024) SARS-CoV-2 variant of concern fitness and adaptation in primary human airway epithelia. Cell Reports 43:1

Ogando NS, Dalebout TJ, Zevenhoven-Dobbe JC, Limpens R, van der Meer Y, Caly L, Druce J, de Vries JJC, Kikkert M, Bárcena M, Sidorov I, Snijder EJ (2020) SARS-coronavirus-2 replication in Vero E6 cells: replication kinetics, rapid adaptation and cytopathology. Journal of General Virology 101:925

Araujo DB, Machado RRG, Amgarten DE, Malta FD, de Araujo GG, Monteiro CO, Candido ED, Soares CP, de Menezes FG, Pires ACC, Santana RAF, Viana AD, Dorlass E, Thomazelli L, Ferreira LCD, Botosso VF, Carvalho CRG, Oliveira DBL, Pinho JRR, Durigon EL (2020) SARS-CoV-2 isolation from the first reported patients in Brazil and establishment of a coordinated task network. Memorias Do Instituto Oswaldo Cruz 115:1

Pohl MO, Busnadiego I, Kufner V, Glas I, Karakus U, Schmutz S, Zaheri M, Abela I, Trkola A, Huber M, Stertz S, Hale BG (2021) SARS-CoV-2 variants reveal features critical for replication in primary human cells. Plos Biology 19:1

Feng XL, Yu DD, Zhang M, Li XH, Zou QC, Ma WT, Han JB, Xu L, Yang CX, Qu W, Deng ZH, Long JY, Long YHP, Li MK, Yao YG, Dong XQ, Zeng JX, Li MH (2022) Characteristics of replication and pathogenicity of SARS-CoV-2 Alpha and Delta isolates. Virologica Sinica 37:804

Chen DY, Turcinovic J, Feng SC, Kenney DJ, Chin CV, Choudhary MC, Conway HL, Semaan M, Close BJ, Tavares AH, Seitz S, Khan N, Kapell S, Crossland NA, Li JZ, Douam F, Baker SC, Connor JH, Saeed M (2023) Article Cell culture systems for isolation of SARS-CoV-2 clinical isolates and generation of recombinant virus. Iscience 26:1

Savellini GG, Anichini G, Cusi MG (2023) SARS-CoV-2 omicron sub-lineages differentially modulate interferon response in human lung epithelial cells. Virus Research 332:1

Touret F, Luciani L, Baronti C, Cochin M, Driouich JS, Gilles M, Thirion L, Nougairède A, de Lamballerie X (2021) Replicative Fitness of a SARS-CoV-2 20I/501Y.V1 Variant from Lineage B.1.1.7 in Human Reconstituted Bronchial Epithelium. Mbio 12:1
