# Supplementary material for: Near Neutral Selectionist Theories (NNST) for SARS-CoV-2 suggested by the substitution-mutation ratio (c/µ) analysis
Source: PLoS One. 2026 Mar 4;21(3):e0343410. doi: 10.1371/journal.pone.0343410 (PMC12959723; doi:10.1371/journal.pone.0343410)
Supplement: S2 Table — See Figures in S6 and S8 Figs for the timeline slopes of these segments. (PDF) [file pone.0343410.s002.pdf]

Table S2. **Linear regression values for non-molecular clock segments** Time-based total genomic substitution rate slope and *c* R^2 values for each dataset and averaged over each dataset for all SARS-CoV-2 segments not exhibiting strict molecular clock, in order of decreasing average R^2. See Figures in S6_Figure and S8_Figure for the timeline slopes of these segments.

Seg	A1a	A1b	A1c	Average	A1a	A1b	A1c	Average	Gene/Protein Function
(NT Length)	*c*	*c*	*c*	*c*	*c* R^2	*c* R^2	*c* R^2	*c* R^2	
**Nsp5 (916)**	2.70	2.60	3.20	2.83±0.32	0.3292	0.3835	0.3642	0.5965	Main protease; cleaves Orf1ab
**N 5'UTR (14)**	24.7	28.3	29.7	27.60±2.58	0.4308	0.5526	0.5792	0.5577	n/a
**M 5'UTR (50)**	1.7	2.2	2.2	2.03±0.29	0.5214	0.4577	0.3572	0.4925	n/a
**Orf8 5'UTR (134)**	8	8.7	8.4	8.37±0.35	0.3674	0.4441	0.3789	0.4862	n/a
**Nsp14 (1,036)**	2.40	2.40	2.30	2.37±0.06	0.3313	0.3439	0.2159	0.4841	Proofreading exonuclease
**Orf8 TRS-B (7)**	6.4	9.8	12	9.40±2.82	0.2621	0.2476	0.1945	0.4698	Transcription initiation for Orf8
**Orf7a (366)**	8.40	8.90	8.50	8.60±0.27	0.3617	0.3533	0.3634	0.4031	Downregulates antiviral activity
**Orf10 (117)**	4.40	4.30	4.40	4.37±0.06	0.3049	0.1001	-0.1541	0.3557	n/a
**Orf7a TRS-B (7)**	10.6	8.7	7.6	8.97±1.52	0.405	0.1256	0.214	0.3129	Transcription initiation for Orf7a
**Nsp7 (247)**	2.40	2.30	2.30	2.33±0.06	0.0458	0.2681	0.0594	0.3018	Cofactor of NSP11/RdRp
**Orf6 5'UTR (10)**	1.4	0.7	1.9	1.33±0.60	0.0394	0.2266	0.1103	0.2871	n/a
**Orf1ab TRS-L (7)**	0	0.2	1.4	0.53±0.76	N/A	0.0705	0.0721	0.2699	Transcription initiation for Orf1ab
**Orf7a 5'UTR (6)**	12.4	10.1	8.9	10.50±1.78	0.405	0.1256	0.214	0.2692	n/a
**N TRS-B (7)**	1.1	1.1	0.8	1.00±0.17	0.3242	0.273	0.1538	0.2535	Transcription initiation for N
**Orf10 5'UTR (24)**	12.4	11.6	8.4	10.80±2.12	-0.0078	0.2689	-0.023	0.2323	n/a
**Orf10 3'UTR (229)**	12.8	12.7	15.4	13.60±1.53	0.4686	0.4604	0.1487	0.1415	Stabilizes viral genome
**E 5'UTR (24)**	1.2	1.5	1.5	1.40±0.17	0.0984	-0.1452	0.0876	0.0399	n/a
**S 5'UTR (7)**	0.7	0.2	0.3	0.40±0.27	-0.0246	0.0433	0.0705	0.0239	n/a
**Orf6 (186)**	2.90	2.80	3.10	2.93±0.15	-0.3076	-0.3942	0.1115	**0**	Inhibits nucleus molecular trafficking
**S TRS-B (7)**	0.7	0	0	0.23±0.40	-0.0246	N/A	N/A	-0.0075	Transcription initiation for S
**Orf3a 5'UTR (8)**	0	0	0.3	0.10±0.17	N/A	N/A	-0.0254	-0.0235	n/a
**Orf3a TRS-B (7)**	0.7	0.7	0.4	0.60±0.17	-0.0254	-0.0181	-0.0254	-0.0402	Transcription initiation for Orf3a
**Orf6 TRS-B (6)**	0.8	1.6	0.4	0.93±0.61	-0.0781	-0.146	-0.0919	-0.211	Transcription initiation for Orf6
**E TRS-B (6)**	0	0	0	0.00±0.00	N/A	N/A	N/A	N/A	Transcription initiation for E
**M TRS-B (7)**	0	0	0	0.00±0.00	N/A	N/A	N/A	N/A	Transcription initiation for M

c = xE-03% / NT site / month.
