# Supplementary material for: Near Neutral Selectionist Theories (NNST) for SARS-CoV-2 suggested by the substitution-mutation ratio (c/µ) analysis
Source: PLoS One. 2026 Mar 4;21(3):e0343410. doi: 10.1371/journal.pone.0343410 (PMC12959723; doi:10.1371/journal.pone.0343410)
Supplement: S3 Table — Time-based total genomic substitution rate slope and c R2 values for each dataset and averaged over each dataset for all SARS-CoV-2 segments exhibiting strict molecular clock, in order of decreasing average R2. See Figures in S7 and S9 Figs for the timeline slopes of these segments. (PDF) [file pone.0343410.s003.pdf]

Table S3. **Linear regression values for molecular clock segments**. Time-based total genomic substitution rate slope and c R^2 values for each dataset and averaged over each dataset for all SARS-CoV-2 segments exhibiting strict molecular clock, in order of decreasing average R^2. See Figures in S7_Figure and S9_Figure for the timeline slopes of these segments.

Seg (NT Length)	A1a *c*	A1b *c*	A1c *c*	Average *c*	A1a *c R²*	A1b *c R²*	A1c *c R²*	Average *c R²*	Gene/Protein Function
**Genome (29,903)**	6.40	6.90	6.50	6.60±0.26	0.9829	0.9775	0.9888	**0.9957**	Encodes virus components
**Orf1ab (21,291)**	4.00	4.00	4.10	4.03±0.06	0.9839	0.9865	0.9862	**0.9854**	Polyprotein; contains NSP1-15
**All-TR (29,133)**	6.10	6.10	6.10	6.10±0.00	0.9790	0.9805	0.9847	**0.9805**	Protein-coding region
**Nsp3 (5,388)**	5.00	5.00	5.00	5.00±0.00	0.9722	0.9610	0.9762	**0.9801**	3CL-like protease; cleaves ORF1ab
**Nsp12 (1,801)**	2.80	2.90	2.80	2.83±0.06	0.9246	0.9067	0.9349	**0.9507**	Helicase; unwinds viral RNA for transcription
**Orf1ab 5'UTR (265)**	35.4	35.9	36.9	36.10±0.76	0.6703	0.6229	0.6556	**0.9392**	Directs viral mRNA to ribosome
**N (1,260)**	23.20	23.3	23.00	2.32±0.15	0.9395	0.9164	0.9213	**0.9497**	Nucleocapsid; virus packing & self-assembly
**Nsp11 (2,794)**	6.30	6.30	6.20	6.27±0.06	0.8975	0.8756	0.8903	**0.9169**	RdRp; synthesizes RNA genome
**S (3,822)**	10.10	10.40	10.20	10.20±0.15	0.8773	0.8909	0.8912	**0.8711**	Glycoprotein; introduces host cell infection
**Nsp2 (1,912)**	4.00	4.00	4.00	4.00±0.00	0.6489	0.6803	0.6747	**0.8517**	Promotes viral RNA synthesis & translation
**Nsp8 (592)**	1.30	1.30	1.50	1.37±0.12	0.7176	0.6084	0.5228	**0.8214**	Cofactor & stabilizer of NSP11
**Nsp9 (337)**	4.30	4.60	4.40	4.43±0.15	0.7794	0.8354	0.7812	**0.7971**	Binds viral ssRNA for nuclear transport
**Nsp10 (415)**	1.30	1.20	1.10	1.20±0.10	0.5713	0.5635	0.7183	**0.7922**	Cofactor & stimulator of NSP13 & NSP14
**M (669)**	4.80	5.00	4.90	4.90±0.10	0.7723	0.7128	0.6655	**0.7902**	Membrane; virus assembly/budding
**E (228)**	2.70	2.80	2.70	2.73±0.06	0.4663	0.5390	0.4510	**0.7201**	Envelope; virus assembly/release, ion channel
**All UTR (771)**	18.5	18.8	19.8	19.00±0.68	0.8024	0.8024	0.6912	**0.7167**	Encodes UTR
**Nsp13 (1,579)**	2.70	2.70	2.80	2.73±0.06	0.7399	0.7188	0.7001	**0.6755**	Exoribonuclease; proofreading machinery
**Nsp1 (538)**	3.00	3.10	3.20	3.10±0.10	0.3726	0.3449	0.4774	**0.6735**	Promotes viral mRNA translation
**Nsp4 (1,498)**	3.90	4.00	4.10	4.00±0.10	0.5536	0.5724	0.5630	**0.6555**	Mediates building of replication organelle
**Nsp15 (892)**	2.10	2.00	2.30	2.13±0.15	0.5331	0.5512	0.4786	**0.6361**	2'-O-methyltransferase; immune evasion
**All TRS (61)**	2.3	2.5	2.6	2.47±0.15	0.4473	0.0471	0.4406	**0.6299**	Encodes TRS
**Orf8 (366)**	22.20	21.90	22.40	22.20±0.25	0.5974	0.5679	0.5866	**0.6135**	Modulates viral replication/host immunity
**Orf3a (828)**	8.60	8.70	9.00	8.77±0.21	0.5892	0.3726	0.6957	**0.6116**	Inhibits autophagy & apoptosis
**Nsp6 (868)**	5.30	5.40	5.10	5.27±0.15	0.5072	0.5249	0.5684	**0.6016**	Mediates building of replication organelle

c = xE-03% / NT site / month.

μ = total Orf1ab 5'UTR substitution rate = 36.61E-03% substitutions / NT site / month.
