# Supplementary material for: Near Neutral Selectionist Theories (NNST) for SARS-CoV-2 suggested by the substitution-mutation ratio (c/µ) analysis
Source: PLoS One. 2026 Mar 4;21(3):e0343410. doi: 10.1371/journal.pone.0343410 (PMC12959723; doi:10.1371/journal.pone.0343410)
Supplement: S4 Table — Time-based c/µ (c/µa), Position-based c/µ (c/µb), the absolute and percent differences for c/µ for the genome, All-UTR, All-TR and each coding segment of SARS-CoV-2 not exhibiting strict molecular clock, in order of decreasing average R2, averaged over the three datasets. (PDF) [file pone.0343410.s004.pdf]

Table S4. Relative rate values for non-molecular clock segments. Time-based *c/μ* (c/μ^a), Position-based *c/μ* (c/μ^b), the absolute and percent differences for *c/μ* for the genome, All-UTR, All-TR and each coding segment of SARS-CoV-2 not exhibiting strict molecular clock, in order of decreasing average R^2, averaged over the three datasets.

Seg (NT Length)	c/μ^a	c/μ^b	|Abs Difference|	|%Difference|	c R^2
**Nsp5 (916)**	0.09(L-)	0.07(L-)	0.02	23.08	0.5965
**N 5'UTR (14)**	0.90(L-)	0.77(L-)	0.13	16.99	0.5577
**M 5'UTR (50)**	0.06(L-)	0.06(L-)	0.01	9.68	0.4925
**Orf8 5'UTR (134)**	0.23(L-)	0.23(L-)	0	0.79	0.4862
**Nsp14 (1,036)**	0.07(L-)	0.06(L-)	0.01	15.15	0.4841
**Orf8 TRS-B (7)**	0.27(L-)	0.26(L-)	0.01	4.93	0.4698
**Orf7a (366)**	0.16(L-)	0.26(L-)	0.1	38.57	0.4031
**Orf10 (117)**	0.14(L-)	0.11(L-)	0.03	31.03	0.3557
**Orf7a TRS-B (7)**	0.29(L-)	0.25(L-)	0.04	15.44	0.3129
**Nsp7 (247)**	0.07(L-)	0.05(L-)	0.02	30	0.3018
**Orf6 5'UTR (10)**	0.03(L-)	0.04(L-)	0.01	15	0.2871
**Orf1ab TRS-L (7)**	0.01(L-)	0.01(L-)	0.01	50	0.2699
**Orf7a 5'UTR (6)**	0.34(L-)	0.29(L-)	0.05	15.72	0.2692
**N TRS-B (7)**	0.03(L-)	0.03(L-)	0.01	26.67	0.2535
**Orf10 5'UTR (24)**	0.27(L-)	0.30(L-)	0.03	9.76	0.2323
**Orf10 3'UTR (229)**	0.38(L-)	0.38(L-)	0	0.48	0.1415
**E 5'UTR (24)**	0.05(L-)	0.04(L-)	0.01	19.05	0.0399
**S 5'UTR (7)**	0.01(L-)	0.01(L-)	0	16.67	0.0239
**Orf6 (186)**	0.09(H-)	0.07(H-)	0.02	23.68	**0**
**S TRS-B (7)**	0.01(L-)	0.01(L-)	0	0	-0.0075
**Orf3a 5'UTR (8)**	0.00(L-)	0.00(L-)	0	50	-0.0235
**Orf3a TRS-B (7)**	0.01(L-)	0.02(L-)	0.01	55.56	-0.0402
**Orf6 TRS-B (6)**	0.03(L-)	0.03(L-)	0.01	21.43	-0.211
**E TRS-B (6)**	0.00(L-)	0.00(L-)	0	0	N/A
**M TRS-B (7)**	0.00(L-)	0.00(L-)	0	0	N/A

*Low coefficient of determination causes significant deviation in c/μ^a.
