# Supplementary material for: Near Neutral Selectionist Theories (NNST) for SARS-CoV-2 suggested by the substitution-mutation ratio (c/µ) analysis
Source: PLoS One. 2026 Mar 4;21(3):e0343410. doi: 10.1371/journal.pone.0343410 (PMC12959723; doi:10.1371/journal.pone.0343410)
Supplement: S5 Table — Time-based c/µ (c/µa), Position-based c/µ (c/µb), the absolute and percent differences for c/µ for the genome, All-UTR, All-TR and each coding segment of SARS-CoV-2 exhibiting strict molecular clock, in order of decreasing average R2, averaged over the three datasets. (PDF) [file pone.0343410.s005.pdf]

Table S5. Relative rate values for molecular clock segments. Time-based c/μ (c/μ^a), Position-based c/μ (c/μ^b), the absolute and percent differences for c/μ for the genome, All-UTR, All-TR and each coding segment of SARS-CoV-2 exhibiting strict molecular clock, in order of decreasing average R^2, averaged over the three datasets.

Seg (NT Length)	c/μ^a	c/μ^b	|Abs Difference|	|%Difference|	c R^2
**Genome (29,903)**	0.18(H-)	0.18(H-)	0	0	**0.9957**
**Orf1ab (21,291)**	0.11(H-)	0.11(H-)	0	0	**0.9854**
**All-TR (29,133)**	0.17(H-)	0.17(H-)	0.01	3.16	**0.9805**
**Nsp3 (5,388)**	0.14(H-)	0.14(H-)	0	1.28	**0.9801**
**Nsp12 (1,801)**	0.08(H-)	0.08(H-)	0	2.33	**0.9507**
**N (1,260)**	0.66(H-)	0.66(H-)	0	0.55	**0.9497**
**Nsp11 (2,794)**	0.18(H-)	0.18(H-)	0	0	**0.9169**
**S (3,822)**	0.28(H-)	0.31(H-)	0.02	7.74	**0.8711**
**Nsp2 (1,912)**	0.12(H-)	0.11(H-)	0.01	6.56	**0.8517**
**Nsp8 (592)**	0.04(H-)	0.04(H-)	0.01	15	**0.8214**
**Nsp9 (337)**	0.12(H-)	0.13(H-)	0.01	9.72	**0.7971**
**Nsp10 (415)**	0.03(H-)	0.03(H-)	0	5.88	**0.7922**
**M (669)**	0.12(H-)	0.13(H-)	0.01	5.63	**0.7902**
**E (228)**	0.08(H-)	0.07(H-)	0.01	12.5	**0.7201**
**All-UTR (771)**	0.51(H-)	0.52(H-)	0.01	2.12	**0.7167**
**Nsp13 (1,579)**	0.07(H-)	0.07(H-)	0	2.63	**0.6755**
**Nsp1 (538)**	0.10(H-)	0.08(H-)	0.01	18.18	**0.6735**
**Nsp4 (1,498)**	0.09(H-)	0.11(H-)	0.02	19.35	**0.6555**
**Nsp15 (892)**	0.06(H-)	0.05(H-)	0.01	20.69	**0.6361**
**All-TRS (61)**	0.07(H-)	0.07(H-)	0	5	**0.6299**
**Orf8 (366)**	0.68(H-)	0.69(H-)	0	0.53	**0.6135**
**5Orf3a (828)**	0.24(H-)	0.23(H-)	0.02	7.26	**0.6116**
**Nsp6 (868)**	0.12(H-)	0.15(H-)	0.02	16.25	**0.6016**

*Low coefficient of determination causes significant deviation in c/μ^a.
