# Supplementary material for: Near Neutral Selectionist Theories (NNST) for SARS-CoV-2 suggested by the substitution-mutation ratio (c/µ) analysis
Source: PLoS One. 2026 Mar 4;21(3):e0343410. doi: 10.1371/journal.pone.0343410 (PMC12959723; doi:10.1371/journal.pone.0343410)
Supplement: S6 Table — Segments are in order of decreasing average R2. (Column 1): Relative abundance of sites under WN, WP and SP selection. (Column 2): Abundance of sites under NN and P selection. (Column 3): Abundance of sites under SN, NN and SP selection. (Column 4): Abundance of sites under WN and SP selection. (Column 5): Abundance of sites under N and P selection. See Fig 4 and Figure of S15 Fig for graphical representations. (PDF) [file pone.0343410.s006.pdf]

Table S6. Abundance of sites under different selection types for non-molecular clock segments. Segments are in order of decreasing average R^2. (Column 1): Relative abundance of sites under *WN*, *WP* and *SP* selection. (Column 2): Abundance of sites under *NN* and *P* selection. (Column 3): Abundance of sites under *SN*, *NN* and *SP* selection. (Column 4): Abundance of sites under *WN* and *SP* selection. (Column 5): Abundance of sites under *N* and *P* selection. See **Fig 4** and Figure of S15_Figure for graphical representations.

Seg	%WN	%WP	%SP	%NN	%P	%SN	%NN	%SP	%WN	%SP	%N	%P
**Nsp5**	83.31	16.69	0.00	100.00	0.00	92.81	7.19	0.00	100.00	0.00	98.80	1.20
**N 5'UTR**	NA	NA	NA	NA	NA	100.00	0.00	0.00	NA	NA	100.00	0.00
**M 5'UTR**	NA	NA	NA	NA	NA	100.00	0.00	0.00	NA	NA	100.00	0.00
**Orf8 5'UTR**	96.41	3.59	0.00	100.00	0.00	79.10	20.90	0.00	100.00	0.00	99.25	0.75
**Nsp14**	88.59	11.41	0.00	100.00	0.00	94.12	5.87	0.00	100.00	0.00	99.32	0.67
**Orf8 TRS-B**	50.00	50.00	0.00	100.00	0.00	71.43	28.58	0.00	100.00	0.00	85.72	14.29
**Orf7a**	96.87	3.13	0.00	100.00	0.00	73.77	26.23	0.00	100.00	0.00	99.18	0.82
**Orf10**	76.96	23.04	0.00	100.00	0.00	88.89	11.11	0.00	100.00	0.00	97.44	2.56
**Orf7a TRS-B**	50.00	50.00	0.00	100.00	0.00	71.43	28.58	0.00	100.00	0.00	85.72	14.29
**Nsp7**	78.51	21.49	0.00	100.00	0.00	94.38	5.63	0.00	100.00	0.00	98.80	1.21
**Orf6 5'UTR**	100.00	0.00	0.00	100.00	0.00	90.00	10.00	0.00	100.00	0.00	100.00	0.00
**Orf1ab TRS-L**	NA	NA	NA	NA	NA	100.00	0.00	0.00	NA	NA	100.00	0.00
**Orf7a 5'UTR**	50.00	50.00	0.00	100.00	0.00	66.67	33.34	0.00	100.00	0.00	83.34	16.67
**N TRS-B**	NA	NA	NA	NA	NA	100.00	0.00	0.00	NA	NA	100.00	0.00
**Orf10 5'UTR**	85.70	14.30	0.00	100.00	0.00	70.83	29.17	0.00	100.00	0.00	95.83	4.17
**Orf10 3'UTR**	87.07	12.93	0.00	100.00	0.00	62.88	37.11	0.00	100.00	0.00	95.19	4.80
**E 5'UTR**	NA	NA	NA	NA	NA	100.00	0.00	0.00	NA	NA	100.00	0.00
**S 5'UTR**	NA	NA	NA	NA	NA	100.00	0.00	0.00	NA	NA	100.00	0.00
**Orf6**	78.73	21.27	0.00	100.00	0.00	92.43	7.57	0.00	100.00	0.00	98.39	1.61
**S TRS-B**	NA	NA	NA	NA	NA	100.00	0.00	0.00	NA	NA	100.00	0.00
**Orf3a 5'UTR**	NA	NA	NA	NA	NA	100.00	0.00	0.00	NA	NA	100.00	0.00
**Orf3a TRS-B**	NA	NA	NA	NA	NA	100.00	0.00	0.00	NA	NA	100.00	0.00
**Orf6 TRS-B**	NA	NA	NA	NA	NA	100.00	0.00	0.00	NA	NA	100.00	0.00
**E TRS-B**	NA	NA	NA	NA	NA	100.00	0.00	0.00	NA	NA	100.00	0.00
**M TRS-B**	NA	NA	NA	NA	NA	100.00	0.00	0.00	NA	NA	100.00	0.00

WN: Weak negative; ***WP***: weak positive; ***SP***: strong positive; ***NN***: near-neutral (***WN* + *WP***); ***SN***: strong negative; ***N***: negative (***SN* + *WN***); ***P***: positive (***SP* + *WP***). The c/μ boundaries for each selection type are defined for each segment in the supporting document and were used to calculate their percent relative abundance.

*NA: Not applicable.
