# Supplementary material for: Near Neutral Selectionist Theories (NNST) for SARS-CoV-2 suggested by the substitution-mutation ratio (c/µ) analysis
Source: PLoS One. 2026 Mar 4;21(3):e0343410. doi: 10.1371/journal.pone.0343410 (PMC12959723; doi:10.1371/journal.pone.0343410)
Supplement: S7 Table — (Column 1): Relative abundance of sites under WN, WP and SP selection. (Column 2): Abundance of sites under NN and P selection. (Column 3): Abundance of sites under SN, NN and SP selection. (Column 4): Abundance of sites under WN and SP selection. (Column 5): Abundance of sites under N and P selection. See Fig 4 and Figure of S16 Fig for graphical representations. (PDF) [file pone.0343410.s007.pdf]

Table S7. Abundance of sites under different selection types for molecular clock segments. (Column 1): Relative abundance of sites under *WN*, *WP* and *SP* selection. (Column 2): Abundance of sites under *NN* and *P* selection. (Column 3): Abundance of sites under *SN*, *NN* and *SP* selection. (Column 4): Abundance of sites under *WN* and *SP* selection. (Column 5): Abundance of sites under *N* and *P* selection. See **Fig 4** and Figure of S16_Figure for graphical representations.

Seg	%WN	%WP	%SP	%NN	%P	%SN	%NN	%SP	%WN	%SP	%N	%P
**Genome**	90.41	8.41	1.19	98.81	9.59	84.21	15.61	0.19	98.69	1.31	98.49	1.52
**All-TR**	90.30	8.38	1.33	98.67	9.70	85.11	14.69	0.20	98.53	1.47	98.55	1.45
**Nsp3**	93.44	6.56	0.00	100.00	6.56	85.90	14.11	0.00	100.00	0.00	99.08	0.93
**Nsp12**	85.33	14.00	0.66	99.34	14.67	91.68	8.27	0.05	99.30	0.70	98.78	1.22
**N**	94.01	5.99	0.00	100.00	5.99	45.64	54.36	0.00	100.00	0.00	96.75	3.25
**Nsp11**	97.39	2.61	0.00	100.00	2.61	80.79	19.21	0.00	100.00	0.00	99.50	0.50
**S**	92.64	7.36	0.00	100.00	7.36	74.05	25.96	0.00	100.00	0.00	98.10	1.91
**Nsp2**	88.59	11.41	0.00	100.00	11.41	88.56	11.45	0.00	100.00	0.00	98.70	1.31
**Nsp8**	85.19	14.81	0.00	100.00	14.81	95.46	4.54	0.00	100.00	0.00	99.33	0.67
**Nsp9**	76.92	23.08	0.00	100.00	23.08	88.50	11.51	0.00	100.00	0.00	97.35	2.66
**Nsp10**	92.85	7.15	0.00	100.00	7.15	96.64	3.36	0.00	100.00	0.00	99.76	0.24
**M**	91.67	8.33	0.00	100.00	8.33	87.44	12.56	0.00	100.00	0.00	98.95	1.05
**E**	89.47	5.26	5.26	94.74	10.53	91.67	7.90	0.44	94.43	5.57	99.13	0.88
**All-UTR**	95.68	4.32	0.00	100.00	4.32	33.98	66.02	0.00	100.00	0.00	97.15	2.85
**Nsp13**	87.82	11.31	0.87	99.13	12.18	92.73	7.21	0.06	99.07	0.93	99.12	0.88
**Nsp1**	88.68	9.43	1.88	98.12	11.32	90.19	9.63	0.18	97.97	2.03	98.89	1.11
**Nsp4**	88.00	11.20	0.80	99.20	12.00	91.67	8.26	0.07	99.05	0.95	99.00	1.00
**Nsp15**	80.39	19.61	0.00	100.00	19.61	93.88	6.12	0.00	100.00	0.00	98.80	1.20
**All-TRS**	51.61	48.39	0.00	100.00	48.39	93.22	6.78	0.00	100.00	0.00	96.72	3.28
**Orf8**	94.85	5.15	0.00	100.00	5.15	36.34	63.66	0.00	100.00	0.00	96.72	3.28
**Orf3a**	90.62	9.38	0.00	100.00	9.38	76.81	23.18	0.00	100.00	0.00	97.82	2.17
**Nsp6**	78.95	21.05	0.00	100.00	21.05	91.26	8.74	0.00	100.00	0.00	98.16	1.84
**Nsp10**	92.85	7.15	0.00	100.00	7.15	96.64	3.36	0.00	100.00	0.00	99.76	0.24

WN: Weak negative; ***WP***: weak positive; ***SP***: strong positive; ***NN***: near-neutral **(*WN* + *WP*)**; ***SN***: strong negative; ***N***: negative **(*SN* + *WN*); *P***: positive **(*SP* + *WP*).** The c/μ boundaries for each selection type are defined for each segment in the supporting document and were used to calculate their percent relative abundance.
