# Supplementary material for: Near Neutral Selectionist Theories (NNST) for SARS-CoV-2 suggested by the substitution-mutation ratio (c/µ) analysis
Source: PLoS One. 2026 Mar 4;21(3):e0343410. doi: 10.1371/journal.pone.0343410 (PMC12959723; doi:10.1371/journal.pone.0343410)
Supplement: S8 Table — The boundaries were defined using Ohta’s approach versus our approach. (PDF) [file pone.0343410.s008.pdf]

Table S8. Mutation fraction types within the SARS-CoV-2 genome, All-TR and All-UTR. The boundaries were defined using Ohta's approach versus our approach.

Seg	c/μ[1]	σ	*WNB, WPB (c/μ)*	*NB (c/μ)*	f^- *(SN)*[c]	f_o^- *(WN)*[c]	f_o *(N)*[c]	f_o^+ *(WP)*[c]	f^+ *(SP)*[c]	f_o^- *(WN)*[d]	f_o^+ *(WP)*[d]	f^+ *(SP)*[d]	$f'_o : f_o$ *(NN:N)*	$f_o^- : f_o^+$ *(WN:WP)*	$f_o^- : f_o^+ + f^+$ *(WN:P)*
Genome[a]	0.18± 0.01	3.01	0.52, 2.02	1.02	97.2	1.31	0.03	0.75	0.77	46.29	26.50	27.21	68.44	0.87	1.76
Genome[b]	0.18± 0.01	N/A	0.05, 19.34	1.02	84.21	14.28	0.03	1.33	0.19	90.38	8.42	1.20	576.70	5.24	9.43
All-TR[a]	0.18± 0.00	3.01	0.52, 2.02	1.02	97.33	1.22	0.03	0.70	0.74	45.86	26.32	27.82	63.89	0.84	1.74
All-TR[b]	0.18± 0.00	N/A	0.05, 19.34	1.02	85.11	13.44	0.03	1.25	0.20	90.26	8.39	1.34	586.56	5.54	9.31
All-UTR[a]	0.51± 0.02	6.82	0.52, 2.02	1.02	93.51	3.63	0.07	1.69	1.17	55.93	26.04	18.03	61.50	1.28	2.15
All-UTR[b]	0.51± 0.02	N/A	0.02, 58.04	1.02	33.98	63.17	0.07	2.85	0.00	95.68	4.32	0.00	715.10	2.15	22.14

**c/μ* and their standard deviations*:* [1] Calculated via dividing the segment substitution rate (*c*) by the approximated mutation rate ($\mu = c$ of Orf1ab 5'UTR), where the standard deviation is sourced from the c/μ values computed across the three genomic datasets.

The fractions of sites under near neutral selection (*fo'***) is equivalent to *c/μ*; the fraction of sites under strict neutral selection (***fo*(*N*)**) is obtained from *c/μ DFE*; the fraction of sites under strong negative selection (**f^-(*SN*),** weak negative selection (**f_o^- (*WN*)),** weak beneficial selection (**f_o^+ (*WP*))** and strong beneficial selection (**f^+(*SP*))** are obtained from the *c/μ DFE***.** Computed fractions are presented as [c] Absolute fractions and [d] Normalized fractions when considering only nearly neutral, strictly neutral and strongly beneficial mutation fractions**. *WNB*:** Weak negative selection boundary; ***NB***: Neutral selection boundary; ***WPB***: Weak beneficial selection boundary**.** ***NN:N****:* The ratio of nearly neutral mutations (***NN*** **=** ***WN*** **+** ***WP***) and strictly neutral mutations (*N*)*;* ***WN:WP****:* The ratio of weakly deleterious mutations and weakly beneficial mutations; ***WN:P***: The ratio of weakly deleterious mutations and weakly beneficial and strongly beneficial mutations (***P*** **=** ***WP*** **+** ***SP***).

Methods for defining the boundaries separating nearly neutral mutations from strongly deleterious and strongly beneficial mutations.

[a]Ohta's method. [b]Our method.
