# Supplementary material for: Near Neutral Selectionist Theories (NNST) for SARS-CoV-2 suggested by the substitution-mutation ratio (c/µ) analysis
Source: PLoS One. 2026 Mar 4;21(3):e0343410. doi: 10.1371/journal.pone.0343410 (PMC12959723; doi:10.1371/journal.pone.0343410)
Supplement: S9 Table — Percentage of UTR, conserved UTR and TR, synonymous mutations and spontaneous mutation rate in the genome of major life forms and their representative species. (PDF) [file pone.0343410.s009.pdf]

Table S9. Percentage of *UTR*, conserved *UTR* and *TR*, synonymous mutations and spontaneous mutation rate in the genome of major life forms and their representative species.

Major Life Form	Representative Species (NT length)	UTR (%)	Conserved UTR(%)	TR (%)	μ (per site per year)
Viruses	SARS-COV-2 (29.9 kB)	2.6	~0.2	97.4	4.7x10^-4 to 2.0 x 10^-2
Bacteria	E. coli (~4.6 Mb)	~10.0	~6.4	~90.0	5.7x10^-6
Fungi	S. cerevisiae (~12 Mb)	25.0	~7.0	75.0	8.3 x 10^-11 to 3.1 x 10^-8
Plants	A. thaliana (~135 Mb)	67.0	~7.0	33.0	2.2x10^-8
Animals	Homo sapiens (~3 bb)	98-99	~3.5	1-2	8.5x10^-11

*This table was sourced from our previous paper.

*Reference: Wu C., Paradis N.J. and Jain K., "Substitution-Mutation Rate Ratio (c/μ) As Molecular Adaptation Test Beyond Ka/Ks: A SARS-COV-2 Case Study", *Journal of Molecular Evolution*. Under review.
