# Supplementary material for: Near Neutral Selectionist Theories (NNST) for SARS-CoV-2 suggested by the substitution-mutation ratio (c/µ) analysis
Source: PLoS One. 2026 Mar 4;21(3):e0343410. doi: 10.1371/journal.pone.0343410 (PMC12959723; doi:10.1371/journal.pone.0343410)
Supplement: S1 Fig — (A). Simplified replication-selection model for a virus population over time under positive selection, neutral selection and negative selection for the first nucleotide position (red circle). A detailed description has been placed in the methods section. Viral genomes are shown as black lines. Mutations are shown as red, green and blue circles. (B). Integrating c/µ per nucleotide site framework with MutSel per codon and Ka/Ks per gene frameworks to quantify the transient scaled selection coefficients (S) across different genome regions. Ne represents the effective haploid population size, Pfix denotes the probability of fixation, and the fitness of a nucleotide mutant (Fj) and wild type (Fi) are provided. (PDF) [file pone.0343410.s010.pdf]

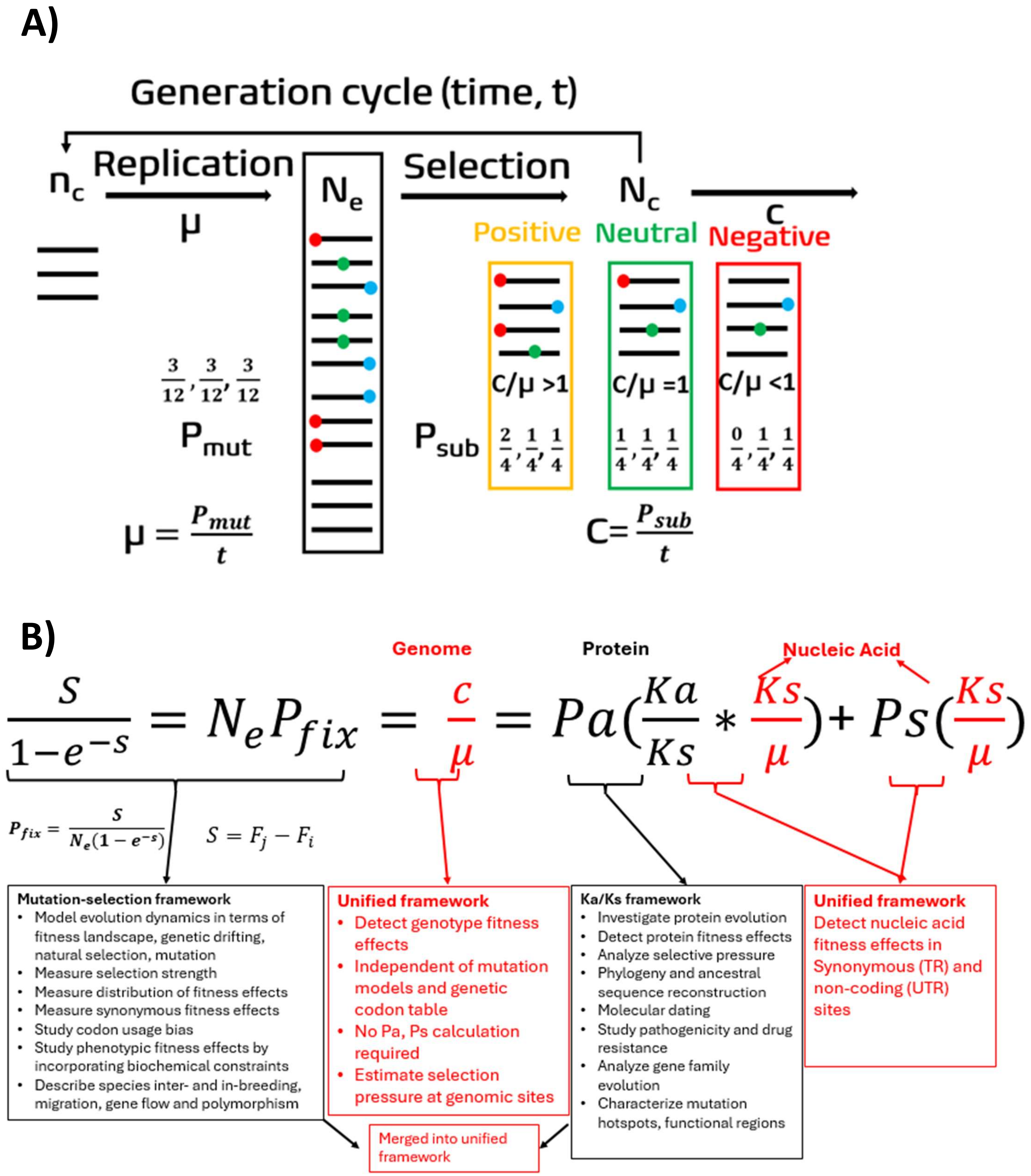

Figure S1. Replication-Selection framework and the linked relationship between *c/μ* framework and the Mutation-Selection framework and *Ka/Ks* framework. (A). Simplified replication-selection model for a virus population over time under positive selection, neutral selection and negative selection for the first nucleotide position (red circle). A detailed

description has been placed in the methods section. Viral genomes are shown as black lines. Mutations are shown as red, green and blue circles. **(B).** Integrating c/μ per nucleotide site framework with MutSel per codon and Ka/Ks per gene frameworks to quantify the transient scaled selection coefficients (S) across different genome regions. N_e represents the effective haploid population size, P_{fix} denotes the probability of fixation, and the fitness of a nucleotide mutant (F_j) and wild type (F_i) are provided.

*These figures were sourced and adapted from our previous paper.

*Reference: Wu C., Paradis N.J. and Jain K., "Substitution-Mutation Rate Ratio (c/μ) As Molecular Adaptation Test Beyond Ka/Ks: A SARS-COV-2 Case Study", *Journal of Molecular Evolution*. Under review.

**Reference: Wu C., Paradis N.J., Lakernick P.M. and Hyrb M., 2023, "L-shaped distribution of the relative substitution rate (c/μ) observed for SARS-COV-2's genome, inconsistent with the selectionist theory, the neutral theory and the nearly neutral theory but a near-neutral balanced selection theory: Implication on "neutralist-selectionist" debate", *Computers in Biology and Medicine*, 153:106522.
