# Supplementary material for: Near Neutral Selectionist Theories (NNST) for SARS-CoV-2 suggested by the substitution-mutation ratio (c/µ) analysis
Source: PLoS One. 2026 Mar 4;21(3):e0343410. doi: 10.1371/journal.pone.0343410 (PMC12959723; doi:10.1371/journal.pone.0343410)
Supplement: S2 Fig — This workflow generates the timelines, SSMRRS and DFE diagrams, approximating µ, calculating c/µ, determining the proportions of different mutation types and evaluating these mutation proportions against five theories of molecular evolutions. (PDF) [file pone.0343410.s011.pdf]

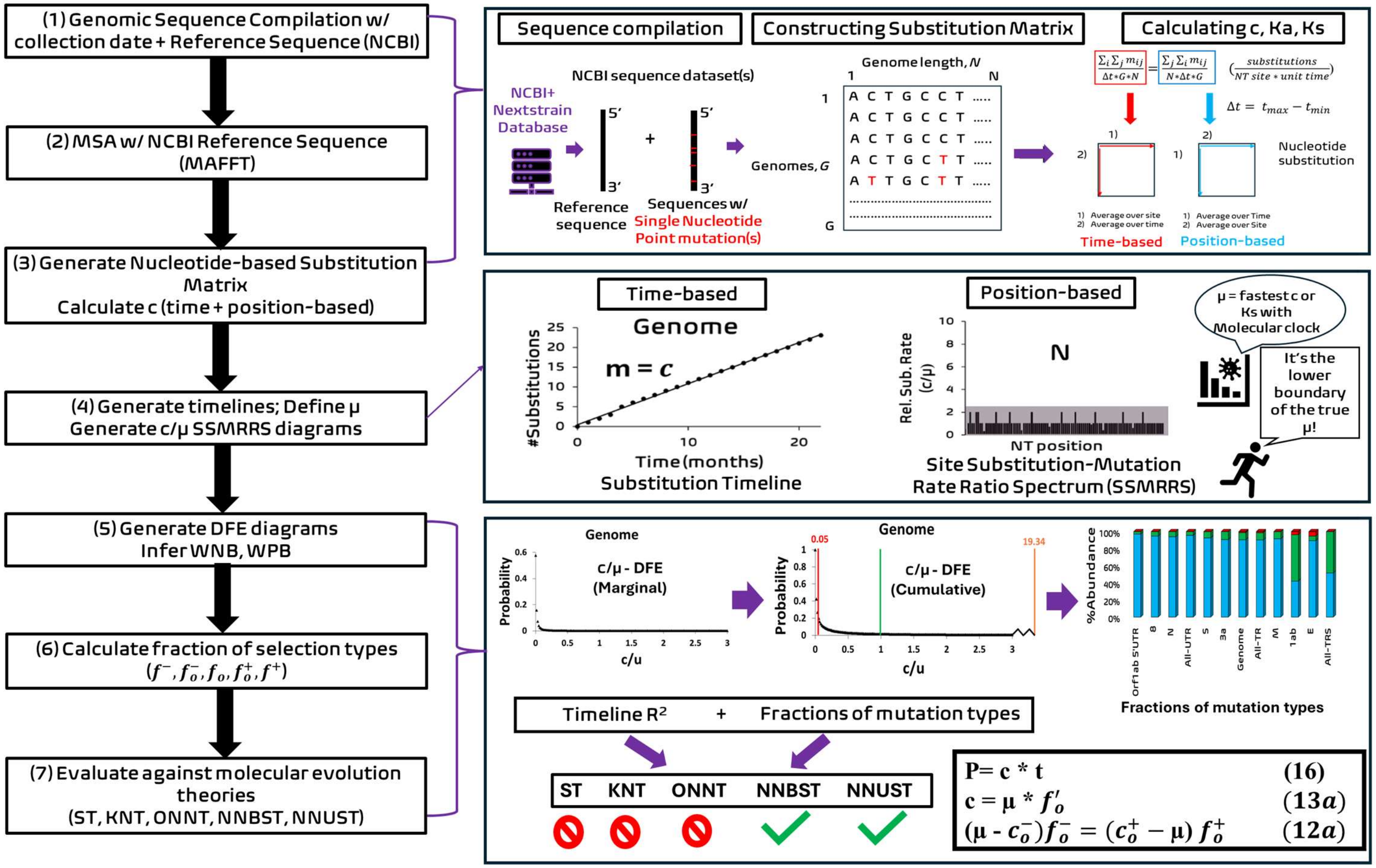

Figure S2. Genomic computational workflow. This workflow generates the timelines, *SSMRRS* and *DFE* diagrams, approximating μ, calculating c/μ, determining the proportions of different mutation types and evaluating these mutation proportions against five theories of molecular evolutions.

*This figure was heavily adapted from our previous papers.

Eq. 16: the integral genetic variance/distance (P) per nucleotide site of a genome at time t is the product of the substitution rate of the whole genome (c) and time. **Eq 13a**: the substitution rate (c) is almost equal to the product of the spontaneous mutation rate (μ) due to sites under near-neutral selection (f_o'). **Eq 12a**: The total substitution rate can be time-independent under the balanced condition of the

Nearly-Neutral Balanced Selectionist Theory (*NNBST*), where the slower, more abundant deleterious mutations $(c^{-}f^{-}, c^{-} < \mu)$ is exactly canceled out by the faster, less abundant advantageous mutations $(c^{+}f^{+}, c^{+} > \mu)$ over the time period.

*Reference: Wu C., Paradis N.J. and Jain K., "Substitution-Mutation Rate Ratio (c/μ) As Molecular Adaptation Test Beyond Ka/Ks: A SARS-COV-2 Case Study", *Journal of Molecular Evolution*. Under review.

** Reference: Paradis N.J. and Wu C., "Enhanced detection and molecular modeling of adaptive mutations in SARS-CoV-2 coding and non-coding regions using the c/μ test", *Virus Evolution*, 10(1):veae089.
