# Supplementary material for: Near Neutral Selectionist Theories (NNST) for SARS-CoV-2 suggested by the substitution-mutation ratio (c/µ) analysis
Source: PLoS One. 2026 Mar 4;21(3):e0343410. doi: 10.1371/journal.pone.0343410 (PMC12959723; doi:10.1371/journal.pone.0343410)
Supplement: S3 Fig — (A) Genome. (B) All-TR. Blue dots represent the c/μ values at each nucleotide and codon site. Orange dots represent the c/μ values of top mutation sites with literature-validated mutation effects. Red dots represent the c/μ values of conserved sites where lethal mutations for SARS-CoV-2 were experimentally-verified by the literature. Black and purple lines represent the optimized lower and upper boundaries for approximating the true μ value. (PDF) [file pone.0343410.s012.pdf]

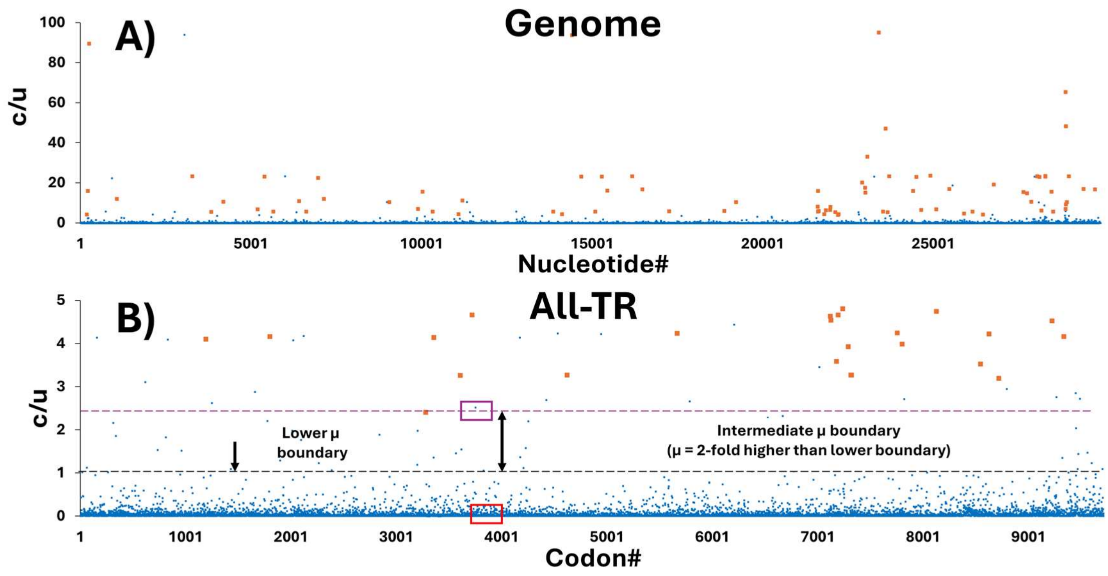

Figure S3. Approximating the true mutation rate (μ) range in a genome and translated coding region. (**A**) Genome. (**B**) All-TR. Blue dots represent the c/μ values at each nucleotide and codon site. Orange dots represent the c/μ values of top mutation sites with literature-validated mutation effects. Red dots represent the c/μ values of conserved sites where lethal mutations for SARS-CoV-2 were experimentally-verified by the literature. Black and purple lines represent the optimized lower and upper boundaries for approximating the true μ value.

* This figure was sourced from our previous paper.

Reference: Chun Wu, Nicholas J. Paradis and Khushi Jain, Substitution-Mutation Rate Ratio (c/μ) As Molecular Adaptation Test Beyond Ka/Ks: A SARS-COV-2 Case Study, Journal of Molecular Evolution. Accepted.
