# Supplementary material for: Near Neutral Selectionist Theories (NNST) for SARS-CoV-2 suggested by the substitution-mutation ratio (c/µ) analysis
Source: PLoS One. 2026 Mar 4;21(3):e0343410. doi: 10.1371/journal.pone.0343410 (PMC12959723; doi:10.1371/journal.pone.0343410)
Supplement: S4 Fig — (A) The structure of the SARS-CoV-2 genome, including major gene segments (Orf1ab, S, E, M and N), accessory genes (Orf3a, Orf6, Orf7a, Orf8 and Orf10), UTRs (Orf1ab 5’-UTR and Orf10 3’-UTR) and TRS (leader TRS-L and TRS-B). (B) Position-based c/µ at each nucleotide site in the SARS-COV-2 genome. (C) Position-based c/µ at each nucleotide site in All-UTR. The red line in S4C Fig represents strict neutral selection (c/µ = 1). µ is the substitution rate of Orf1ab 5’UTR. (PDF) [file pone.0343410.s013.pdf]

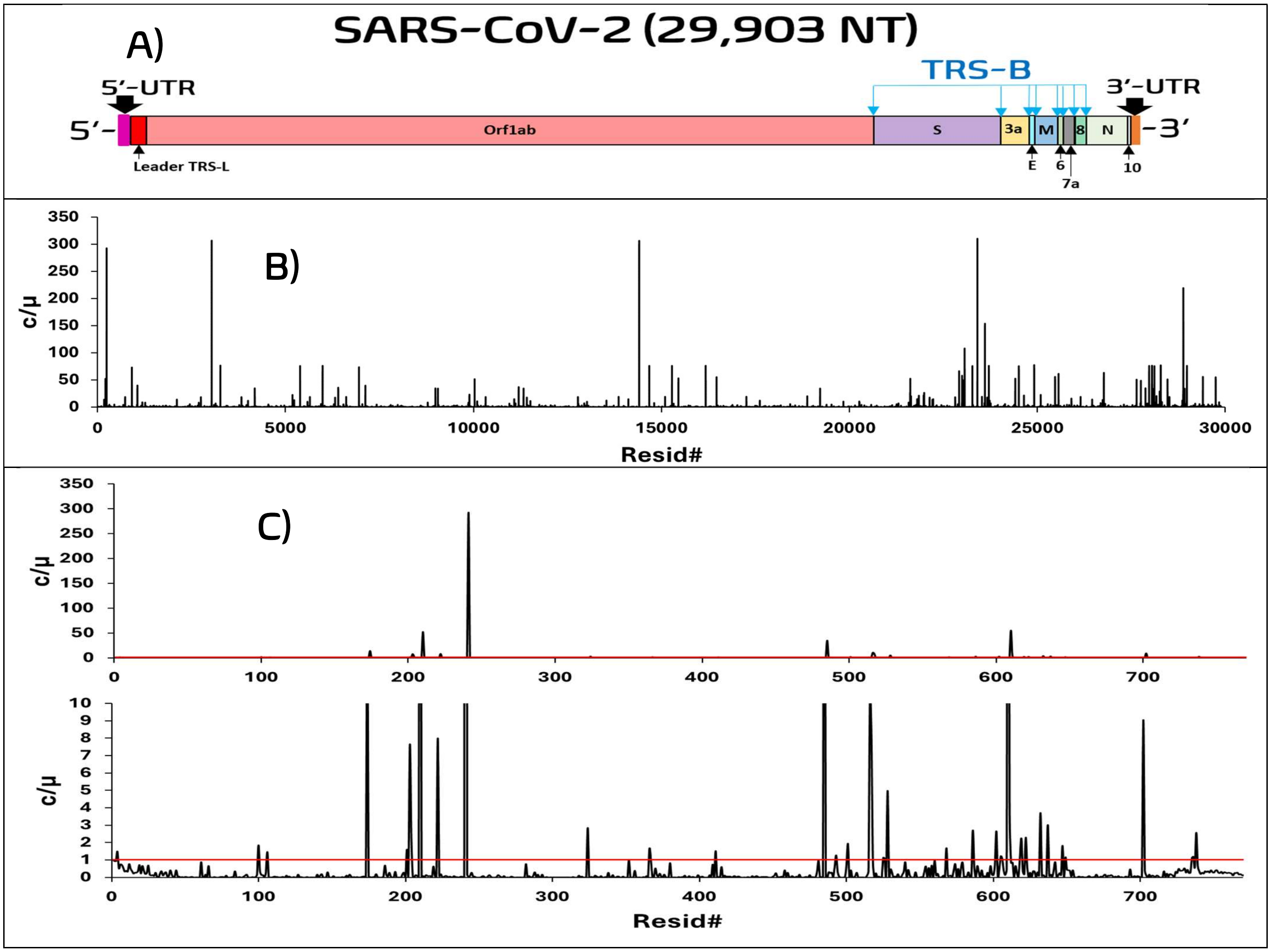

Figure S4. Nucleotide position-based *c/μ* in the SARS-CoV-2 genome. (**A**) The structure of the SARS-CoV-2 genome, including major gene segments (Orf1ab, S, E, M and N), accessory genes (Orf3a, Orf6, Orf7a, Orf8 and Orf10), UTRs (Orf1ab 5'-UTR and Orf10 3'-UTR) and TRS (leader TRS-L and TRS-B). (**B**) Position-based *c/μ* at each nucleotide site in the SARS-COV-2 genome. (**C**) Position-based *c/μ* at each nucleotide site in All-UTR. The red line in Figure S4C represents strict neutral selection ($c/\mu = 1$). μ is the substitution rate of Orf1ab 5'UTR.
