# Supplementary material for: Near Neutral Selectionist Theories (NNST) for SARS-CoV-2 suggested by the substitution-mutation ratio (c/µ) analysis
Source: PLoS One. 2026 Mar 4;21(3):e0343410. doi: 10.1371/journal.pone.0343410 (PMC12959723; doi:10.1371/journal.pone.0343410)
Supplement: S5 Fig — Percent genomic variation of sequences in set A1a with the references sequences Wuhan-Hu-1 (left) and Wuhan IPBCAMS-WH-01 2019 (right) set. (PDF) [file pone.0343410.s014.pdf]

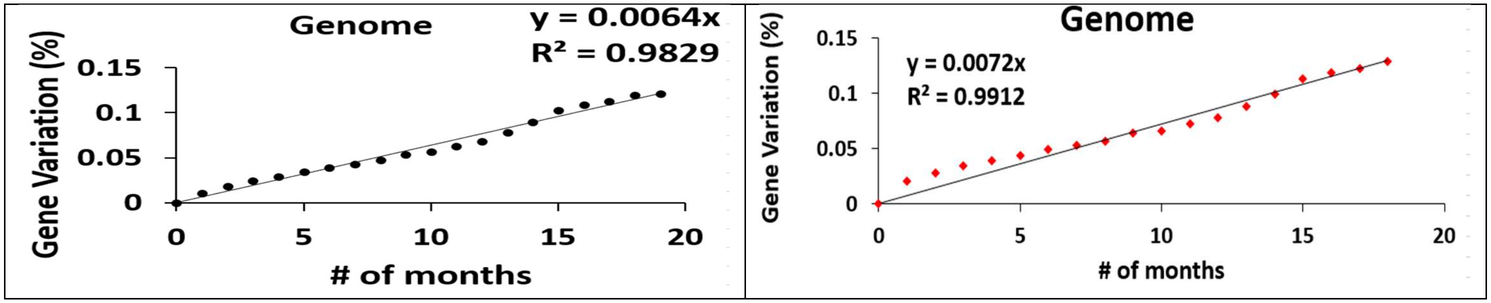

Figure S5. Comparison of molecular clocks using different SARS-CoV-2 ancestral sequences. Percent genomic variation of sequences in set A1a with the references sequences Wuhan-Hu-1 (left) and Wuhan IPBCAMS-WH-01 2019 (right) set.
