# Supplementary material for: Near Neutral Selectionist Theories (NNST) for SARS-CoV-2 suggested by the substitution-mutation ratio (c/µ) analysis
Source: PLoS One. 2026 Mar 4;21(3):e0343410. doi: 10.1371/journal.pone.0343410 (PMC12959723; doi:10.1371/journal.pone.0343410)
Supplement: S6 Fig — The percent total nucleotide substitution rate for the segments not exhibiting strict molecular clock averaged over the three combined datasets, in order of decreasing average R2 (from left to right, top to bottom). See Table of S2 Table for tabulated regression parameters. (PDF) [file pone.0343410.s015.pdf]

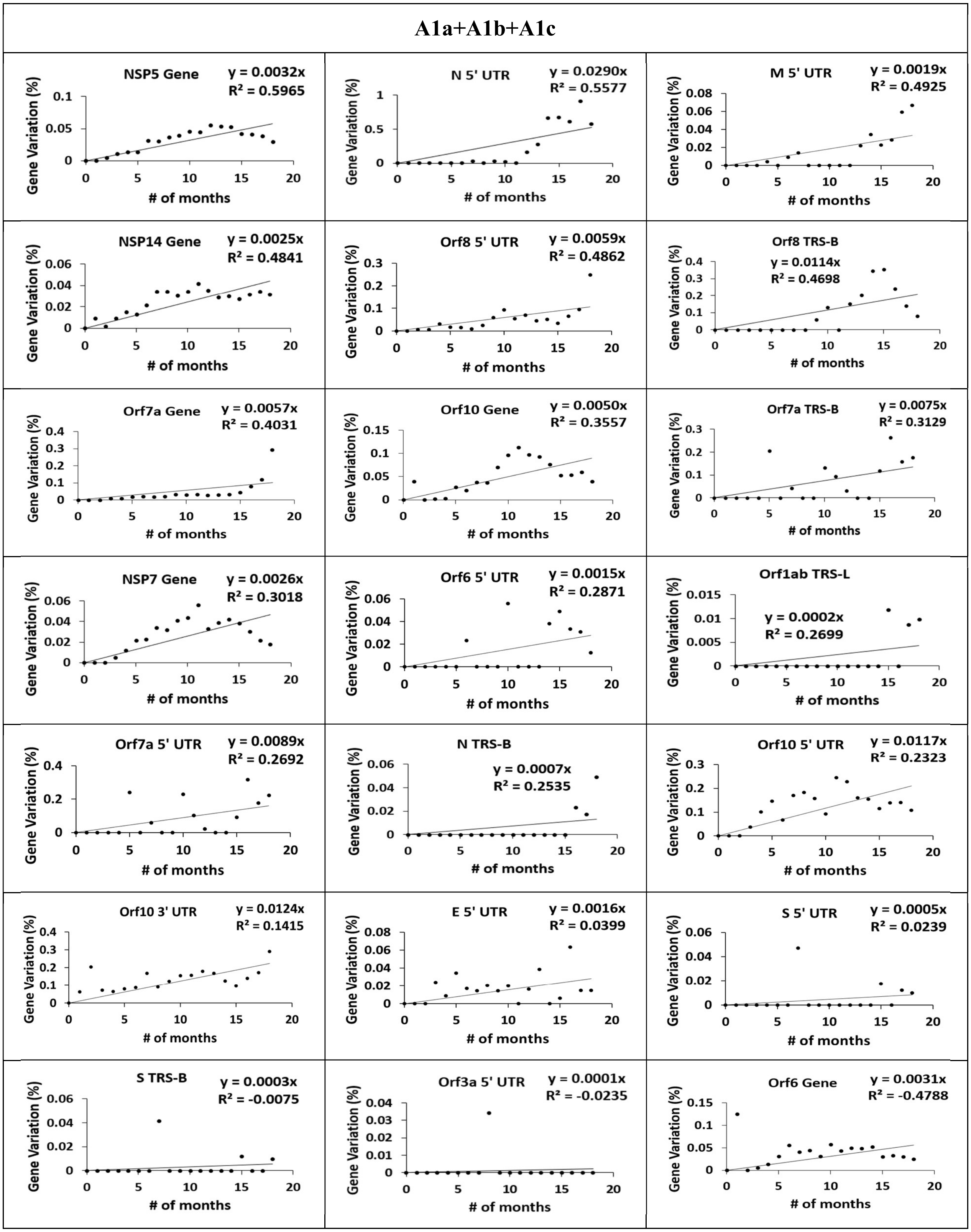
A1a+A1b+A1c
NSP5 Gene
y = 0.0032x
R² = 0.5965
N 5' UTR
y = 0.0290x
R² = 0.5577
M 5' UTR
y = 0.0019x
R² = 0.4925
NSP14 Gene
y = 0.0025x
R² = 0.4841
Orf8 5' UTR
y = 0.0059x
R² = 0.4862
Orf8 TRS-B
y = 0.0114x
R² = 0.4698
Orf7a Gene
y = 0.0057x
R² = 0.4031
Orf10 Gene
y = 0.0050x
R² = 0.3557
Orf7a TRS-B
y = 0.0075x
R² = 0.3129
NSP7 Gene
y = 0.0026x
R² = 0.3018
Orf6 5' UTR
y = 0.0015x
R² = 0.2871
Orf1ab TRS-L
y = 0.0002x
R² = 0.2699
Orf7a 5' UTR
y = 0.0089x
R² = 0.2692
N TRS-B
y = 0.0007x
R² = 0.2535
Orf10 5' UTR
y = 0.0117x
R² = 0.2323
Orf10 3' UTR
y = 0.0124x
R² = 0.1415
E 5' UTR
y = 0.0016x
R² = 0.0399
S 5' UTR
y = 0.0005x
R² = 0.0239
S TRS-B
y = 0.0003x
R² = -0.0075
Orf3a 5' UTR
y = 0.0001x
R² = -0.0235
Orf6 Gene
y = 0.0031x
R² = -0.4788
Gene Variation (%)
# of months

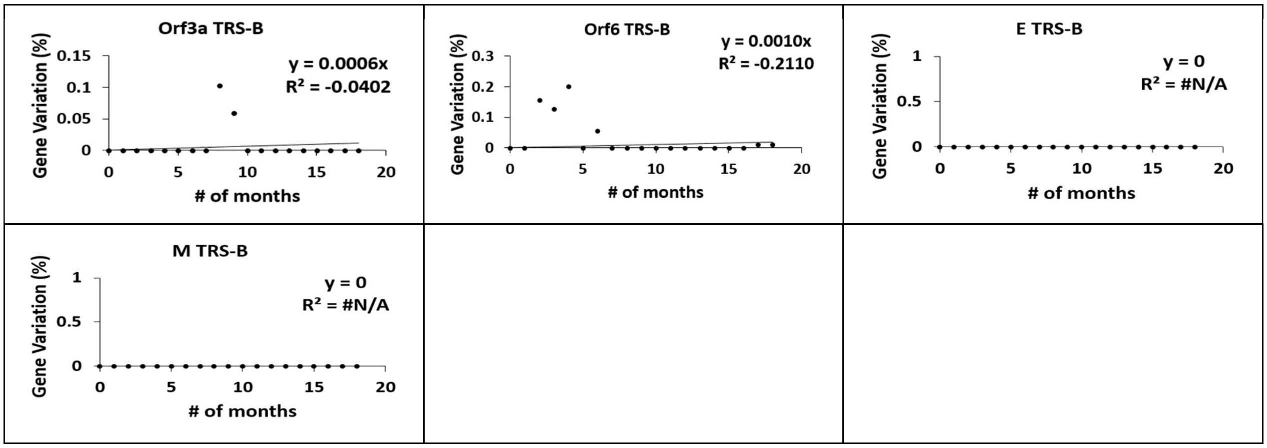

Figure S6. Timelines for non-molecular clock segments across three datasets. The percent total nucleotide substitution rate for the segments not exhibiting strict molecular clock averaged over the three combined datasets, in order of decreasing average R^2 (from left to right, top to bottom)**.** See Table of S2_Table for tabulated regression parameters.
