# Supplementary material for: Near Neutral Selectionist Theories (NNST) for SARS-CoV-2 suggested by the substitution-mutation ratio (c/µ) analysis
Source: PLoS One. 2026 Mar 4;21(3):e0343410. doi: 10.1371/journal.pone.0343410 (PMC12959723; doi:10.1371/journal.pone.0343410)
Supplement: S7 Fig — The percent total nucleotide substitution rate for the segments exhibiting strict molecular clock averaged over the three combined datasets, in order of decreasing average R2 (from left to right, top to bottom). See Table of S3 Table for tabulated regression parameters. (PDF) [file pone.0343410.s016.pdf]

A1a+A1b+A1c

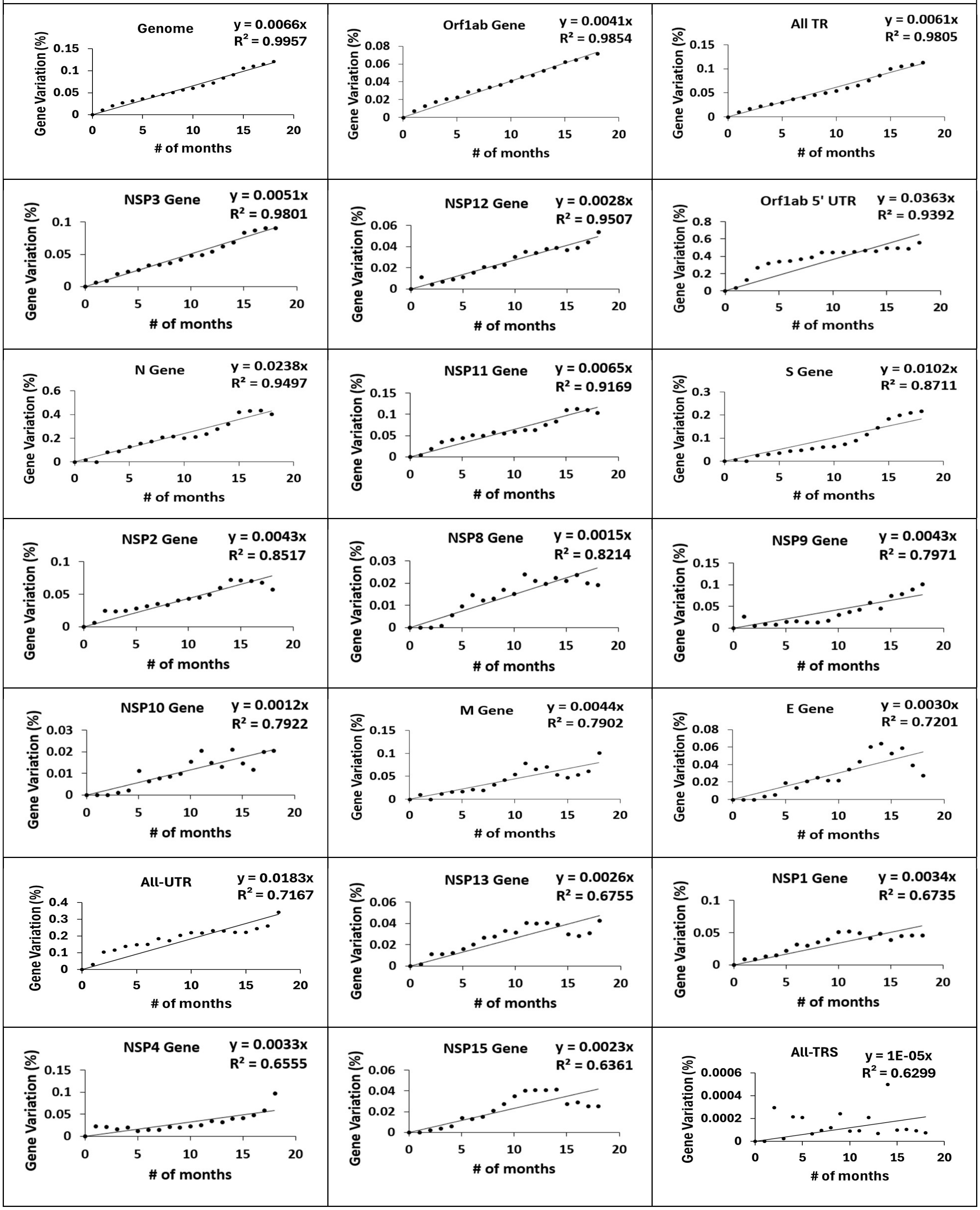

Genome
y = 0.0066x
R² = 0.9957
Orf1ab Gene
y = 0.0041x
R² = 0.9854
All TR
y = 0.0061x
R² = 0.9805
NSP3 Gene
y = 0.0051x
R² = 0.9801
NSP12 Gene
y = 0.0028x
R² = 0.9507
Orf1ab 5' UTR
y = 0.0363x
R² = 0.9392
N Gene
y = 0.0238x
R² = 0.9497
NSP11 Gene
y = 0.0065x
R² = 0.9169
S Gene
y = 0.0102x
R² = 0.8711
NSP2 Gene
y = 0.0043x
R² = 0.8517
NSP8 Gene
y = 0.0015x
R² = 0.8214
NSP9 Gene
y = 0.0043x
R² = 0.7971
NSP10 Gene
y = 0.0012x
R² = 0.7922
M Gene
y = 0.0044x
R² = 0.7902
E Gene
y = 0.0030x
R² = 0.7201
All-UTR
y = 0.0183x
R² = 0.7167
NSP13 Gene
y = 0.0026x
R² = 0.6755
NSP1 Gene
y = 0.0034x
R² = 0.6735
NSP4 Gene
y = 0.0033x
R² = 0.6555
NSP15 Gene
y = 0.0023x
R² = 0.6361
All-TRS
y = 1E-05x
R² = 0.6299
Gene Variation (%)
# of months

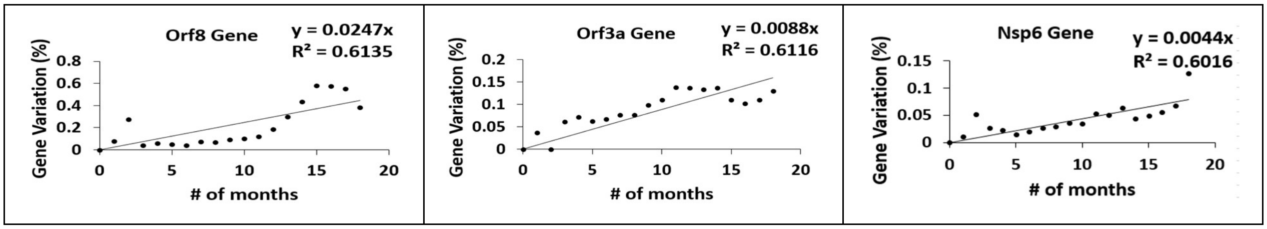

Figure S7. Timelines for molecular clock segments across three datasets. The percent total nucleotide substitution rate for the segments exhibiting strict molecular clock averaged over the three combined datasets, in order of decreasing average R^2 (from left to right, top to bottom)**.** See Table of S3_Table for tabulated regression parameters.
