# Supplementary material for: Near Neutral Selectionist Theories (NNST) for SARS-CoV-2 suggested by the substitution-mutation ratio (c/µ) analysis
Source: PLoS One. 2026 Mar 4;21(3):e0343410. doi: 10.1371/journal.pone.0343410 (PMC12959723; doi:10.1371/journal.pone.0343410)
Supplement: S8 Fig — The percent total nucleotide substitution rate for the segments not exhibiting strict molecular clock for each dataset, in order of decreasing average R2 (from left to right, top to bottom). See Table of S2 Table for tabulated regression parameters. (PDF) [file pone.0343410.s017.pdf]

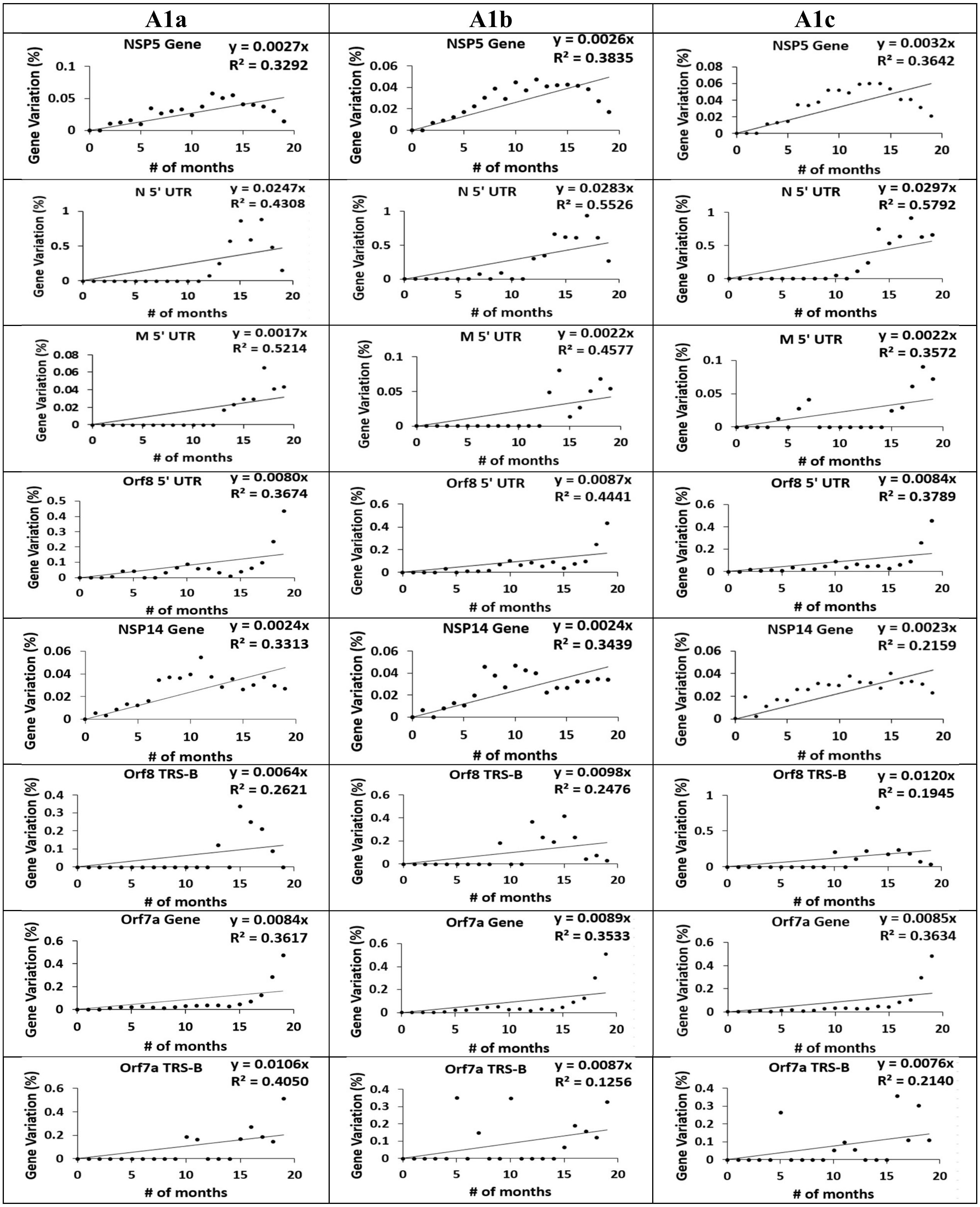
A1a
A1b
A1c
NSP5 Gene
y = 0.0027x
R² = 0.3292
NSP5 Gene
y = 0.0026x
R² = 0.3835
NSP5 Gene
y = 0.0032x
R² = 0.3642
N 5' UTR
y = 0.0247x
R² = 0.4308
N 5' UTR
y = 0.0283x
R² = 0.5526
N 5' UTR
y = 0.0297x
R² = 0.5792
M 5' UTR
y = 0.0017x
R² = 0.5214
M 5' UTR
y = 0.0022x
R² = 0.4577
M 5' UTR
y = 0.0022x
R² = 0.3572
Orf8 5' UTR
y = 0.0080x
R² = 0.3674
Orf8 5' UTR
y = 0.0087x
R² = 0.4441
Orf8 5' UTR
y = 0.0084x
R² = 0.3789
NSP14 Gene
y = 0.0024x
R² = 0.3313
NSP14 Gene
y = 0.0024x
R² = 0.3439
NSP14 Gene
y = 0.0023x
R² = 0.2159
Orf8 TRS-B
y = 0.0064x
R² = 0.2621
Orf8 TRS-B
y = 0.0098x
R² = 0.2476
Orf8 TRS-B
y = 0.0120x
R² = 0.1945
Orf7a Gene
y = 0.0084x
R² = 0.3617
Orf7a Gene
y = 0.0089x
R² = 0.3533
Orf7a Gene
y = 0.0085x
R² = 0.3634
Orf7a TRS-B
y = 0.0106x
R² = 0.4050
Orf7a TRS-B
y = 0.0087x
R² = 0.1256
Orf7a TRS-B
y = 0.0076x
R² = 0.2140
Gene Variation (%)
# of months

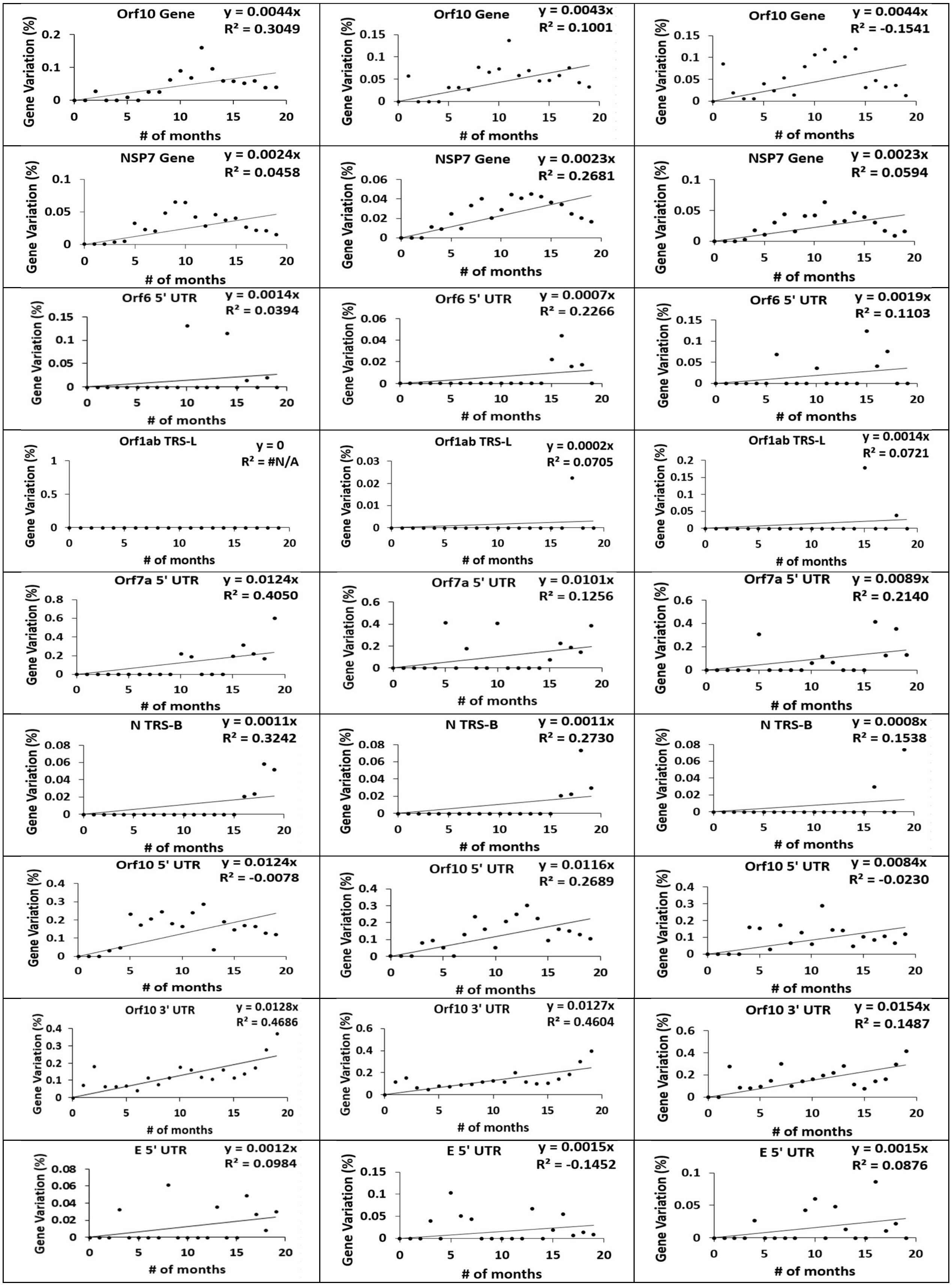
Orf10 Gene
y = 0.0044x
R² = 0.3049
Orf10 Gene
y = 0.0043x
R² = 0.1001
Orf10 Gene
y = 0.0044x
R² = -0.1541
NSP7 Gene
y = 0.0024x
R² = 0.0458
NSP7 Gene
y = 0.0023x
R² = 0.2681
NSP7 Gene
y = 0.0023x
R² = 0.0594
Orf6 5' UTR
y = 0.0014x
R² = 0.0394
Orf6 5' UTR
y = 0.0007x
R² = 0.2266
Orf6 5' UTR
y = 0.0019x
R² = 0.1103
Orf1ab TRS-L
y = 0
R² = #N/A
Orf1ab TRS-L
y = 0.0002x
R² = 0.0705
Orf1ab TRS-L
y = 0.0014x
R² = 0.0721
Orf7a 5' UTR
y = 0.0124x
R² = 0.4050
Orf7a 5' UTR
y = 0.0101x
R² = 0.1256
Orf7a 5' UTR
y = 0.0089x
R² = 0.2140
N TRS-B
y = 0.0011x
R² = 0.3242
N TRS-B
y = 0.0011x
R² = 0.2730
N TRS-B
y = 0.0008x
R² = 0.1538
Orf10 5' UTR
y = 0.0124x
R² = -0.0078
Orf10 5' UTR
y = 0.0116x
R² = 0.2689
Orf10 5' UTR
y = 0.0084x
R² = -0.0230
Orf10 3' UTR
y = 0.0128x
R² = 0.4686
Orf10 3' UTR
y = 0.0127x
R² = 0.4604
Orf10 3' UTR
y = 0.0154x
R² = 0.1487
E 5' UTR
y = 0.0012x
R² = 0.0984
E 5' UTR
y = 0.0015x
R² = -0.1452
E 5' UTR
y = 0.0015x
R² = 0.0876
Gene Variation (%)
# of months

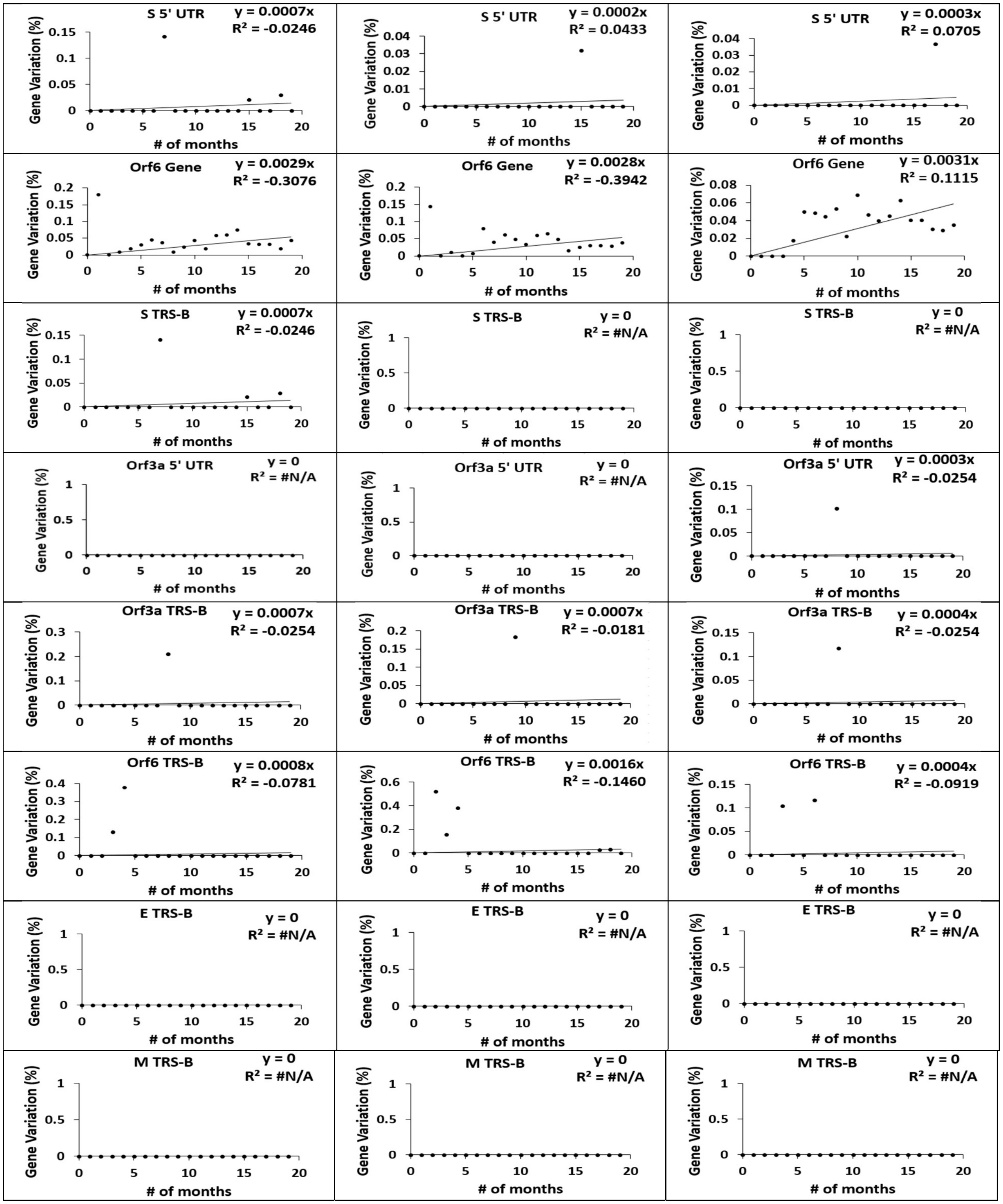

Figure S8. Timelines for non-molecular clock segments across each dataset. The percent total nucleotide substitution rate for the segments not exhibiting strict molecular clock for each dataset, in order of decreasing average R^2 (from left to right, top to bottom)**.** See Table of S2_Table for tabulated regression parameters.
