# Supplementary material for: Near Neutral Selectionist Theories (NNST) for SARS-CoV-2 suggested by the substitution-mutation ratio (c/µ) analysis
Source: PLoS One. 2026 Mar 4;21(3):e0343410. doi: 10.1371/journal.pone.0343410 (PMC12959723; doi:10.1371/journal.pone.0343410)
Supplement: S9 Fig — The percent total nucleotide substitution rate for the segments exhibiting strict molecular clock for each dataset, in order of decreasing average R2 (from left to right, top to bottom). See Table of S3 Table for tabulated regression parameters. (PDF) [file pone.0343410.s018.pdf]

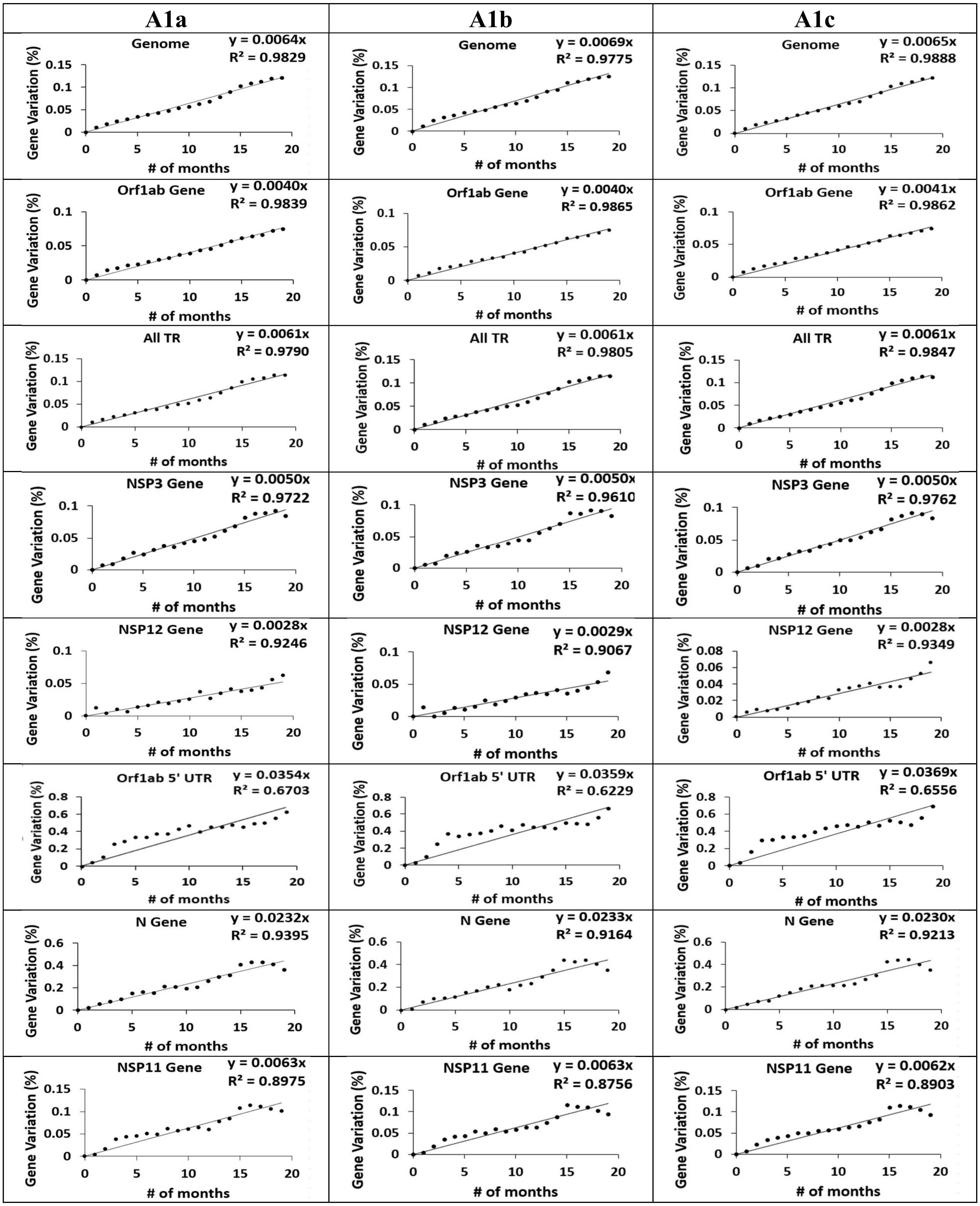
A1a
A1b
A1c
Genome
y = 0.0064x
R² = 0.9829
Genome
y = 0.0069x
R² = 0.9775
Genome
y = 0.0065x
R² = 0.9888
Orf1ab Gene
y = 0.0040x
R² = 0.9839
Orf1ab Gene
y = 0.0040x
R² = 0.9865
Orf1ab Gene
y = 0.0041x
R² = 0.9862
All TR
y = 0.0061x
R² = 0.9790
All TR
y = 0.0061x
R² = 0.9805
All TR
y = 0.0061x
R² = 0.9847
NSP3 Gene
y = 0.0050x
R² = 0.9722
NSP3 Gene
y = 0.0050x
R² = 0.9610
NSP3 Gene
y = 0.0050x
R² = 0.9762
NSP12 Gene
y = 0.0028x
R² = 0.9246
NSP12 Gene
y = 0.0029x
R² = 0.9067
NSP12 Gene
y = 0.0028x
R² = 0.9349
Orf1ab 5' UTR
y = 0.0354x
R² = 0.6703
Orf1ab 5' UTR
y = 0.0359x
R² = 0.6229
Orf1ab 5' UTR
y = 0.0369x
R² = 0.6556
N Gene
y = 0.0232x
R² = 0.9395
N Gene
y = 0.0233x
R² = 0.9164
N Gene
y = 0.0230x
R² = 0.9213
NSP11 Gene
y = 0.0063x
R² = 0.8975
NSP11 Gene
y = 0.0063x
R² = 0.8756
NSP11 Gene
y = 0.0062x
R² = 0.8903
Gene Variation (%)
# of months

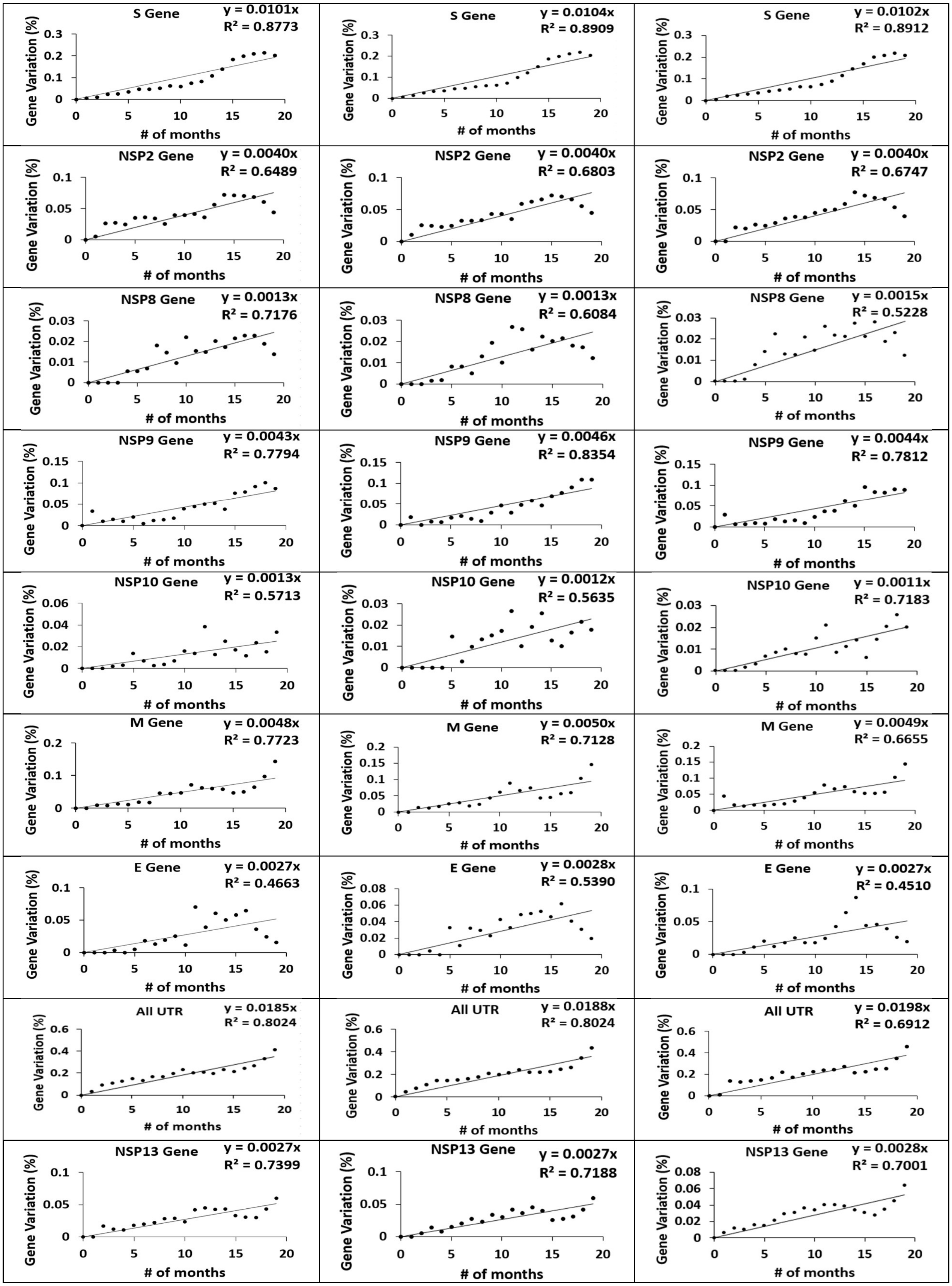
S Gene
y = 0.0101x
R² = 0.8773
S Gene
y = 0.0104x
R² = 0.8909
S Gene
y = 0.0102x
R² = 0.8912
NSP2 Gene
y = 0.0040x
R² = 0.6489
NSP2 Gene
y = 0.0040x
R² = 0.6803
NSP2 Gene
y = 0.0040x
R² = 0.6747
NSP8 Gene
y = 0.0013x
R² = 0.7176
NSP8 Gene
y = 0.0013x
R² = 0.6084
NSP8 Gene
y = 0.0015x
R² = 0.5228
NSP9 Gene
y = 0.0043x
R² = 0.7794
NSP9 Gene
y = 0.0046x
R² = 0.8354
NSP9 Gene
y = 0.0044x
R² = 0.7812
NSP10 Gene
y = 0.0013x
R² = 0.5713
NSP10 Gene
y = 0.0012x
R² = 0.5635
NSP10 Gene
y = 0.0011x
R² = 0.7183
M Gene
y = 0.0048x
R² = 0.7723
M Gene
y = 0.0050x
R² = 0.7128
M Gene
y = 0.0049x
R² = 0.6655
E Gene
y = 0.0027x
R² = 0.4663
E Gene
y = 0.0028x
R² = 0.5390
E Gene
y = 0.0027x
R² = 0.4510
All UTR
y = 0.0185x
R² = 0.8024
All UTR
y = 0.0188x
R² = 0.8024
All UTR
y = 0.0198x
R² = 0.6912
NSP13 Gene
y = 0.0027x
R² = 0.7399
NSP13 Gene
y = 0.0027x
R² = 0.7188
NSP13 Gene
y = 0.0028x
R² = 0.7001
Gene Variation (%)
# of months

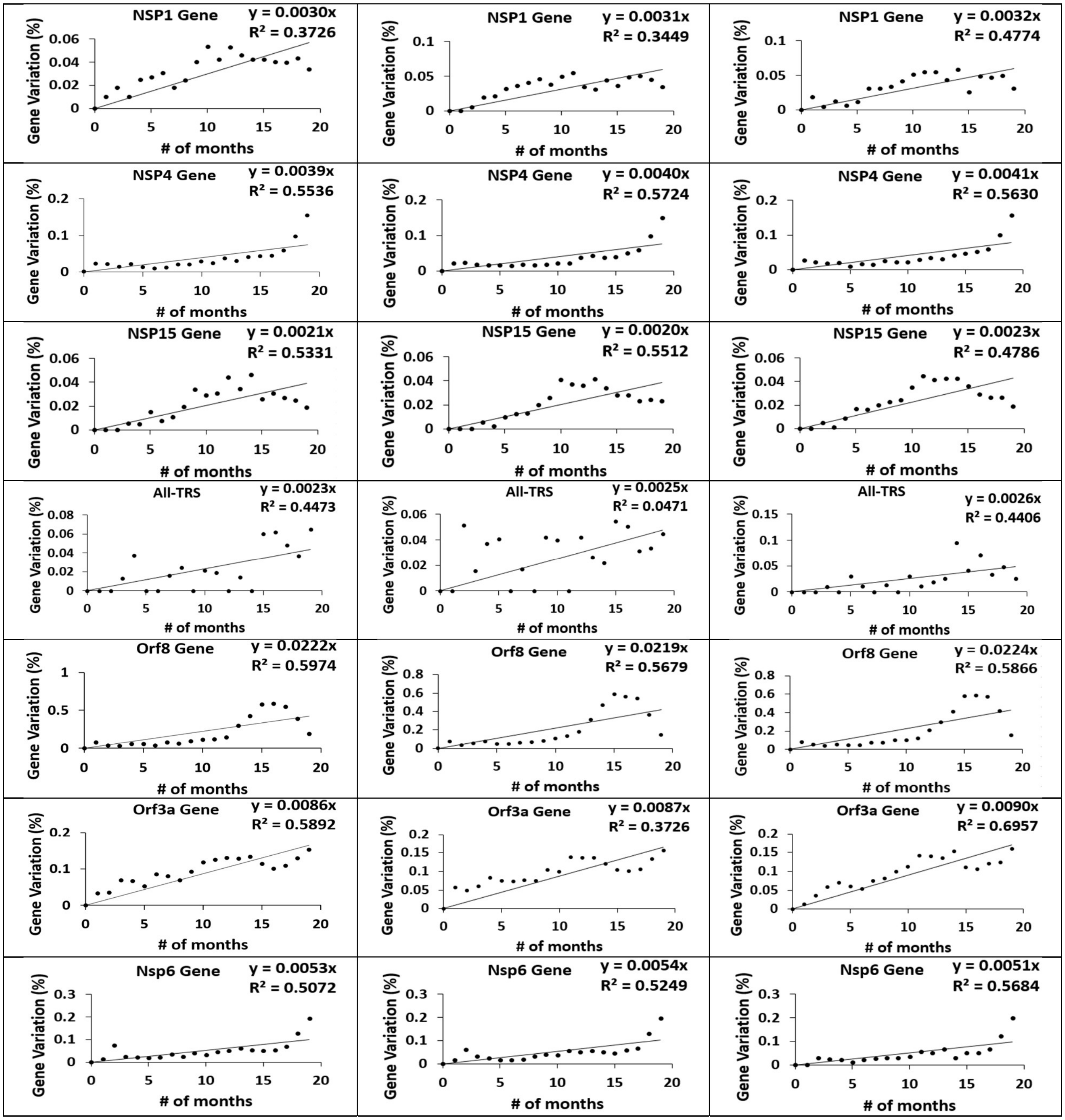

Figure S9. Timelines for molecular clock segments across each dataset. The percent total nucleotide substitution rate for the segments exhibiting strict molecular clock for each dataset, in order of decreasing average R^2 (from left to right, top to bottom)**.** See Table of S3_Table for tabulated regression parameters.
