# Supplementary material for: Near Neutral Selectionist Theories (NNST) for SARS-CoV-2 suggested by the substitution-mutation ratio (c/µ) analysis
Source: PLoS One. 2026 Mar 4;21(3):e0343410. doi: 10.1371/journal.pone.0343410 (PMC12959723; doi:10.1371/journal.pone.0343410)
Supplement: S10 Fig — Monthly percent total nucleotide substitution rate for Orf1ab and NSP1–15 genes over 19 months. NSP segments exhibiting faster or slower substitution rates relative to Orf1ab are under weak beneficial and weak negative selection with respect to Orf1ab (effective neutral selection). (PDF) [file pone.0343410.s019.pdf]

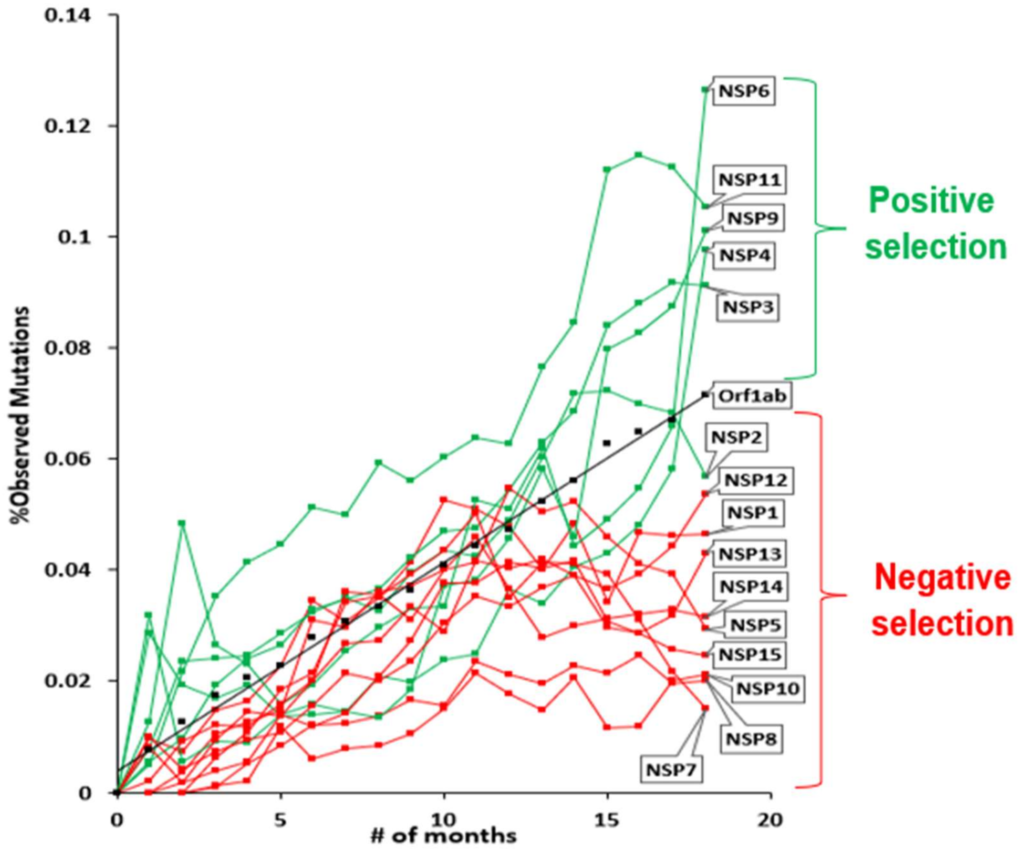

Figure S10. Demonstration of NNBST at the subgenetic level of Orf1ab and NSP1-15. Monthly percent total nucleotide substitution rate for Orf1ab and NSP1-15 genes over 19 months. NSP segments exhibiting faster or slower substitution rates relative to Orf1ab are under weak beneficial and weak negative selection with respect to Orf1ab (effective neutral selection).
