# Supplementary material for: Near Neutral Selectionist Theories (NNST) for SARS-CoV-2 suggested by the substitution-mutation ratio (c/µ) analysis
Source: PLoS One. 2026 Mar 4;21(3):e0343410. doi: 10.1371/journal.pone.0343410 (PMC12959723; doi:10.1371/journal.pone.0343410)
Supplement: S11 Fig — Segments are in order of decreasing average R2 (from left to right, top to bottom), showing the abundance of sites under different selection types. The boundaries for weak negative (red line) to neutral selection (green line) to weak positive selection (orange line) and their determined c/µ positions are noted. Strong negative selection and strong positive selection would be to the left and right of the red and orange lines, respectively. A broken line leading on the x-axis represents the distance between c/µ = 3.0 to the weak positive selection boundary. See Tables 4 and 5 for tabulated c/µ boundaries and percent abundances for each selection type. (PDF) [file pone.0343410.s020.pdf]

A1a/A1b/A1c

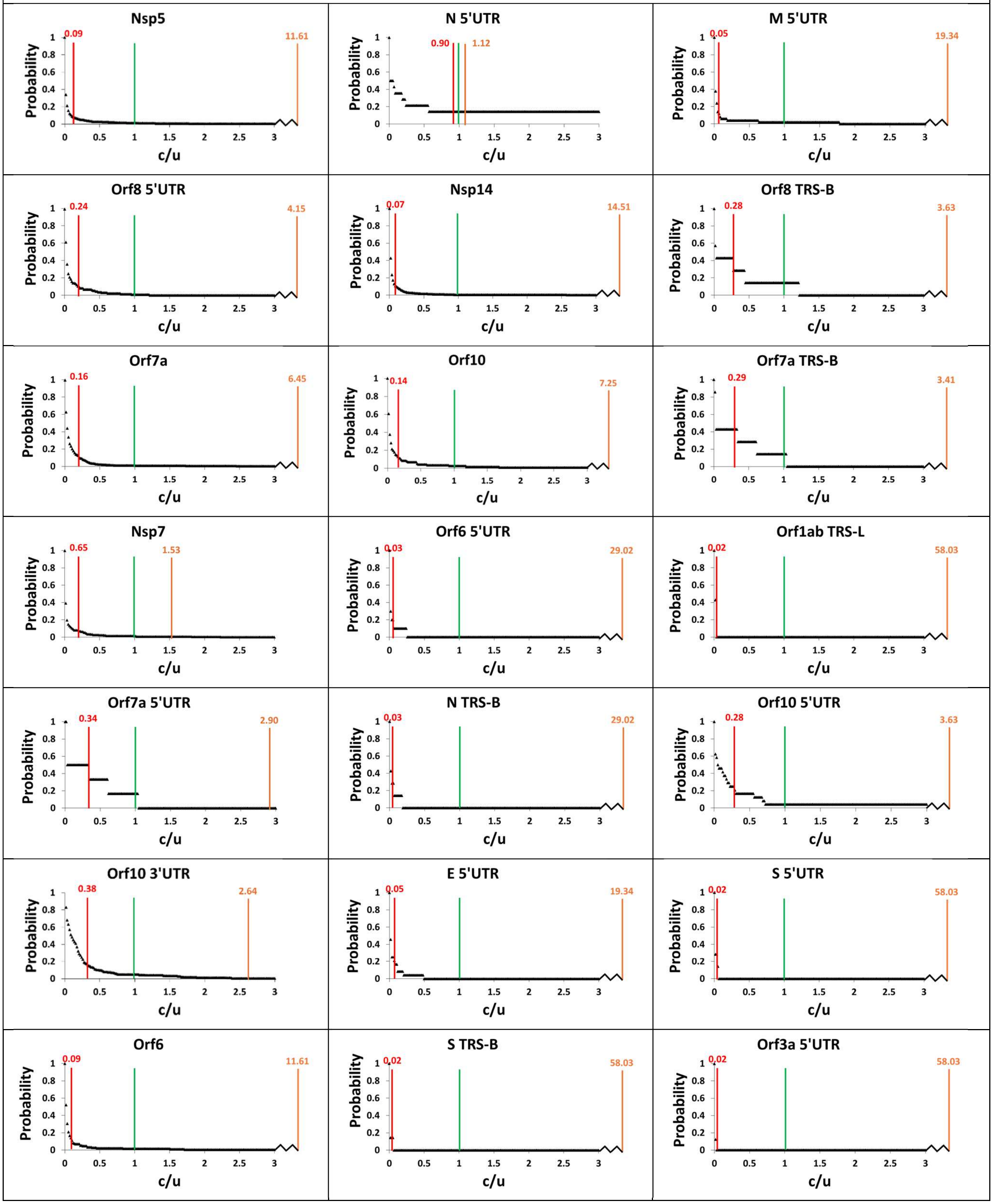

Nsp5
0.09
11.61
N 5'UTR
0.90
1.12
M 5'UTR
0.05
19.34
Orf8 5'UTR
0.24
4.15
Nsp14
0.07
14.51
Orf8 TRS-B
0.28
3.63
Orf7a
0.16
6.45
Orf10
0.14
7.25
Orf7a TRS-B
0.29
3.41
Nsp7
0.65
1.53
Orf6 5'UTR
0.03
29.02
Orf1ab TRS-L
0.02
58.03
Orf7a 5'UTR
0.34
2.90
N TRS-B
0.03
29.02
Orf10 5'UTR
0.28
3.63
Orf10 3'UTR
0.38
2.64
E 5'UTR
0.05
19.34
S 5'UTR
0.02
58.03
Orf6
0.09
11.61
S TRS-B
0.02
58.03
Orf3a 5'UTR
0.02
58.03
Probability
c/u

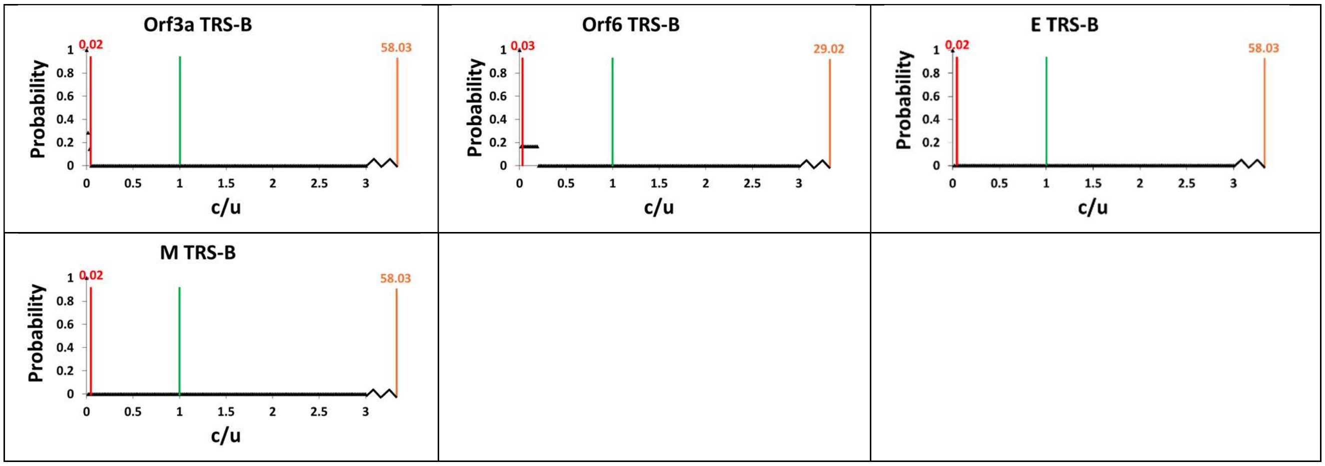

Figure S11. *c/μ* Cumulative probability distribution of non-molecular clock segments. Segments are in order of decreasing average R^2 (from left to right, top to bottom), showing the abundance of sites under different selection types**.** The boundaries for weak negative (red line) to neutral selection (green line) to weak positive selection (orange line) and their determined *c/μ* positions are noted. Strong negative selection and strong positive selection would be to the left and right of the red and orange lines, respectively. A broken line leading on the x-axis represents the distance between *c/μ* = 3.0 to the weak positive selection boundary. See **Table 4** and **Table 5** for tabulated *c/μ* boundaries and percent abundances for each selection type.
