# Supplementary material for: Near Neutral Selectionist Theories (NNST) for SARS-CoV-2 suggested by the substitution-mutation ratio (c/µ) analysis
Source: PLoS One. 2026 Mar 4;21(3):e0343410. doi: 10.1371/journal.pone.0343410 (PMC12959723; doi:10.1371/journal.pone.0343410)
Supplement: S13 Fig — Segments are in order of decreasing average R2 (from left to right, top to bottom), showing the abundance of sites under strictly neutral selection. See Tables 4 and 5 for tabulated percent abundances for strictly neutral selection. (PDF) [file pone.0343410.s022.pdf]

A1a/A1b/A1c

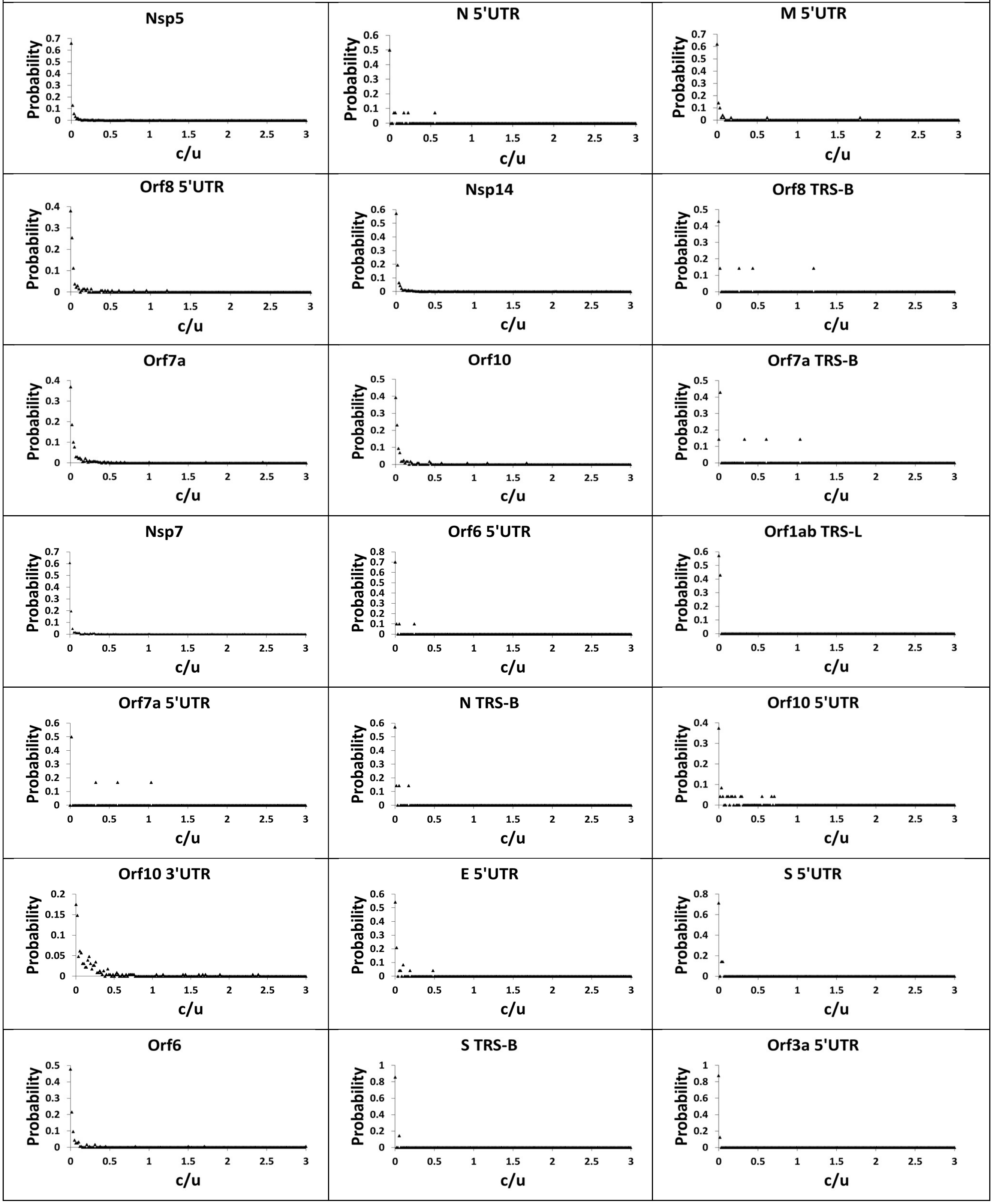

Nsp5
N 5'UTR
M 5'UTR
Orf8 5'UTR
Nsp14
Orf8 TRS-B
Orf7a
Orf10
Orf7a TRS-B
Nsp7
Orf6 5'UTR
Orf1ab TRS-L
Orf7a 5'UTR
N TRS-B
Orf10 5'UTR
Orf10 3'UTR
E 5'UTR
S 5'UTR
Orf6
S TRS-B
Orf3a 5'UTR
Probability
c/u

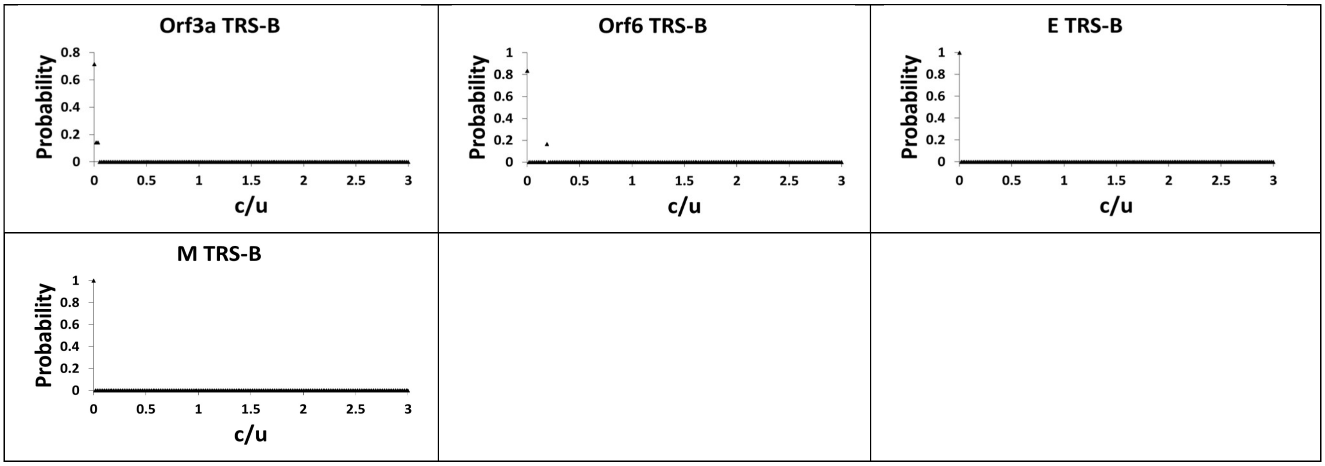

Figure S13. ***c/μ*** **Discrete probability distribution of non-molecular clock segments.** Segments are in order of decreasing average R^2 (from left to right, top to bottom), showing the abundance of sites under strictly neutral selection. See **Table 4** and **Table 5** for tabulated percent abundances for strictly neutral selection.
