# Supplementary material for: Near Neutral Selectionist Theories (NNST) for SARS-CoV-2 suggested by the substitution-mutation ratio (c/µ) analysis
Source: PLoS One. 2026 Mar 4;21(3):e0343410. doi: 10.1371/journal.pone.0343410 (PMC12959723; doi:10.1371/journal.pone.0343410)
Supplement: S14 Fig — Segments are in order of decreasing average R2 (from left to right, top to bottom), showing the abundance of sites under strictly neutral selection. See Tables 4 and 5 for tabulated percent abundances for strictly neutral selection. (PDF) [file pone.0343410.s023.pdf]

A1a/A1b/A1c

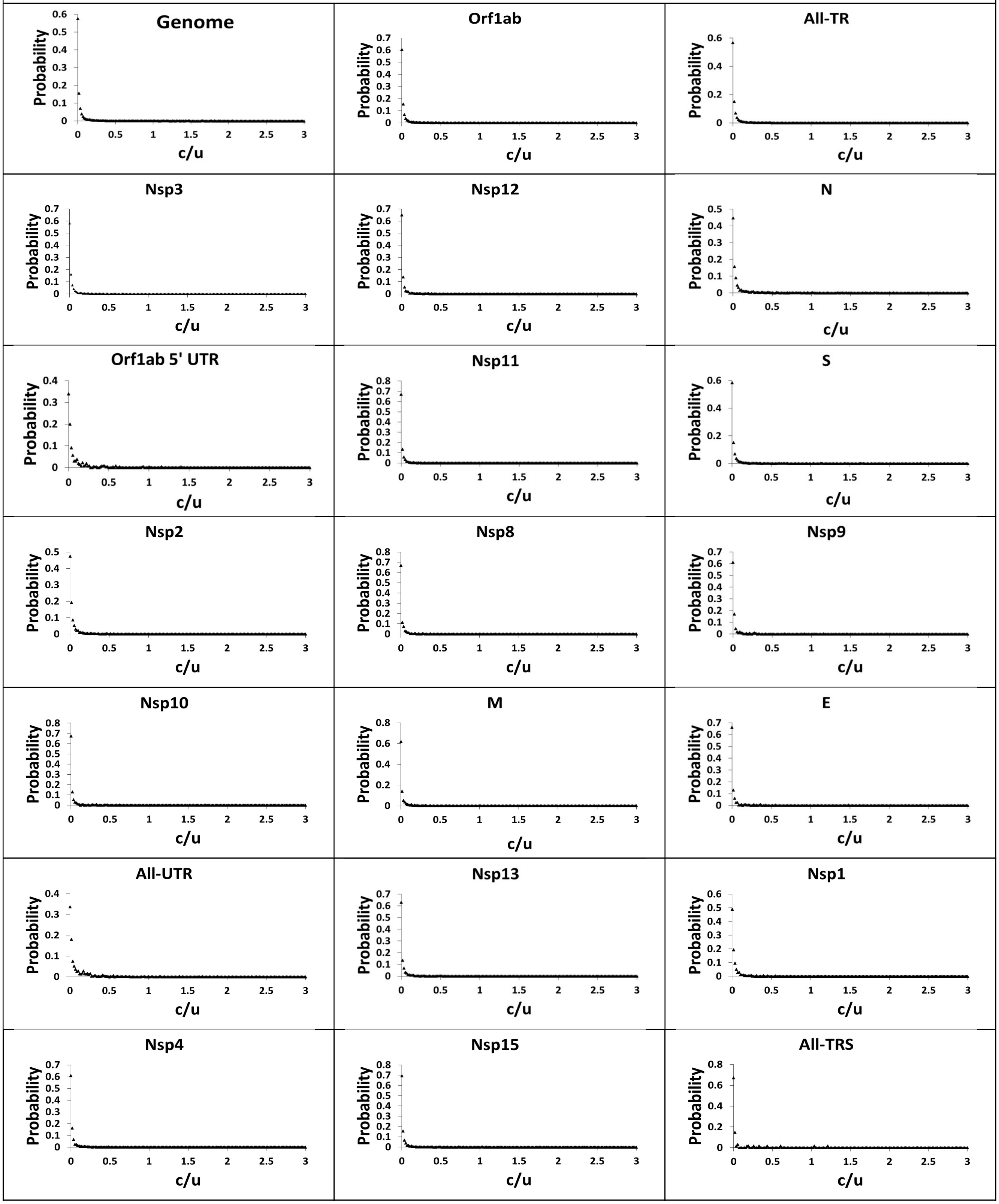
Genome
Orf1ab
All-TR
Nsp3
Nsp12
N
Orf1ab 5' UTR
Nsp11
S
Nsp2
Nsp8
Nsp9
Nsp10
M
E
All-UTR
Nsp13
Nsp1
Nsp4
Nsp15
All-TRS
Probability
c/u

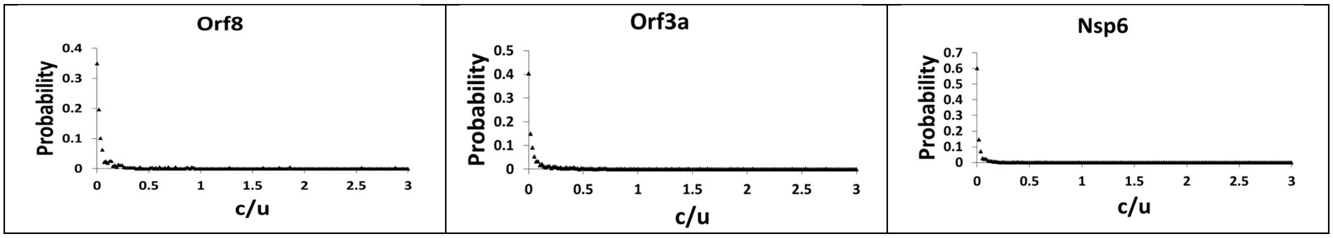

Figure S14. ***c/μ* Discrete probability distribution of molecular clock segments.** Segments are in order of decreasing average R^2 (from left to right, top to bottom), showing the abundance of sites under strictly neutral selection. See **Table 4** and **Table 5** for tabulated percent abundances for strictly neutral selection.
