# Supplementary material for: Near Neutral Selectionist Theories (NNST) for SARS-CoV-2 suggested by the substitution-mutation ratio (c/µ) analysis
Source: PLoS One. 2026 Mar 4;21(3):e0343410. doi: 10.1371/journal.pone.0343410 (PMC12959723; doi:10.1371/journal.pone.0343410)
Supplement: S16 Fig — Selection types are decomposed into three categories for strong negative, near-neutral and strong positive selection (top row), weak negative and strong positive selection (middle row) and negative and positive selection (bottom row) using a c/µ scaling of 5.46 (i.e., lower boundary of true µ). See Table of S7 Table for percent selection type values. (PDF) [file pone.0343410.s025.pdf]

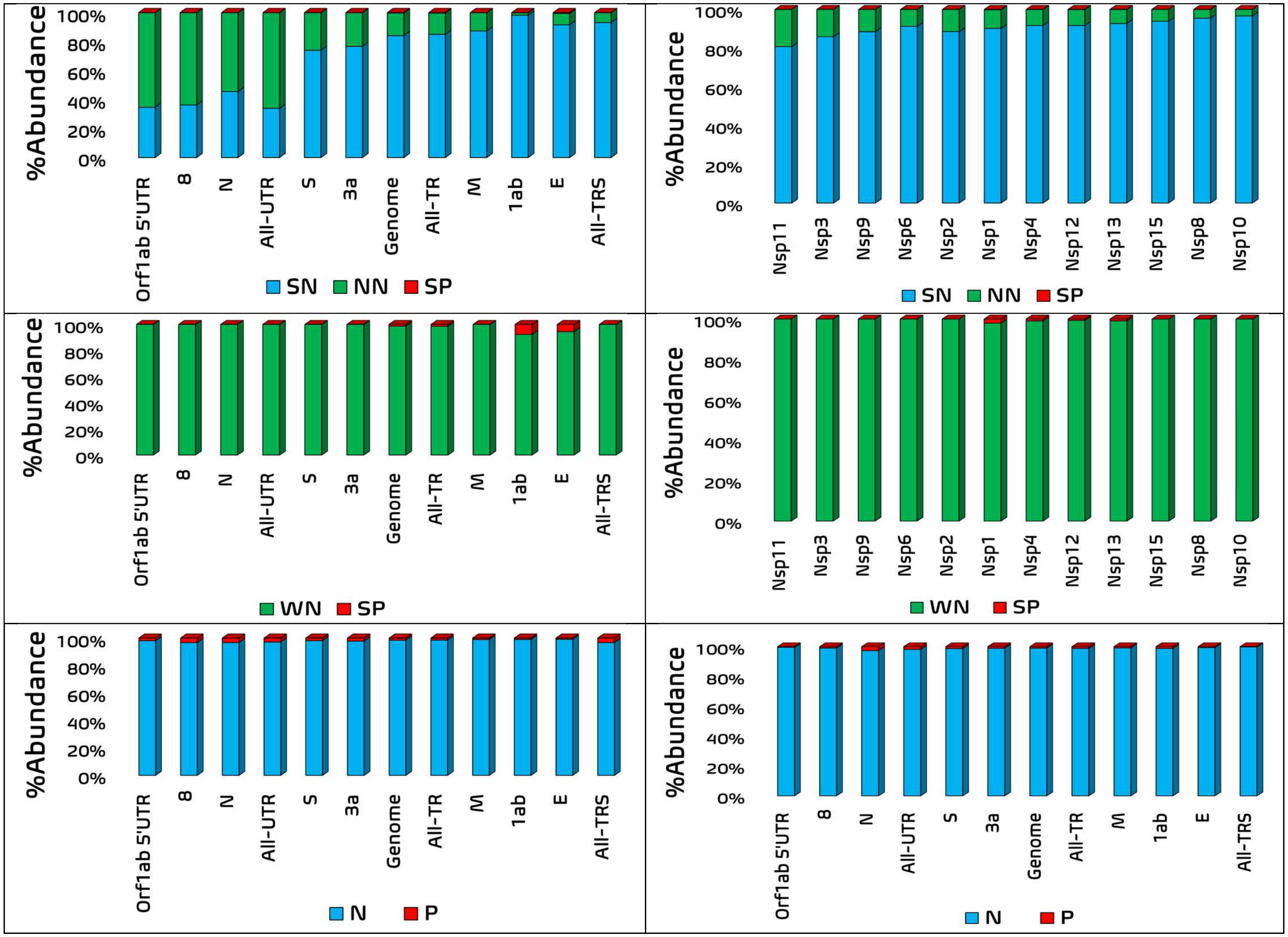

Figure S16. Percent selection types of molecular clock segments. Selection types are decomposed into three categories for strong negative, near-neutral and strong positive selection (top row), weak negative and strong positive selection (middle row) and negative and positive selection (bottom row) using a c/μ scaling of 5.46 (i.e. lower boundary of true μ). See Table of S7_Table for percent selection type values.
