# Supplementary material for: Near Neutral Selectionist Theories (NNST) for SARS-CoV-2 suggested by the substitution-mutation ratio (c/µ) analysis
Source: PLoS One. 2026 Mar 4;21(3):e0343410. doi: 10.1371/journal.pone.0343410 (PMC12959723; doi:10.1371/journal.pone.0343410)
Supplement: S17 Fig — Hg19 phyloP histograms (https://genomewiki.ucsc.edu/index.php/Hg19_phyloP_histograms). Histogram data for phyloP data on the 46-way conservation track on the human genome browser, hg19 each of these data tables have 2,845,303,719 data values. (A) Primate subset (primate subset data statistics: minimum: −9.065, maximum: 0.655, mean: 0.04482, standard deviation: 0.600051); (B) Placental mammal subset (minimum: −13.796, maximum: 2.941, mean: 0.03594, standard deviation: 0.779426), (C). All 46 vertebrates (minimum: −14.08, maximum: 6.424, mean: 0.0896, standard deviation: 0.833186). (PDF) [file pone.0343410.s026.pdf]

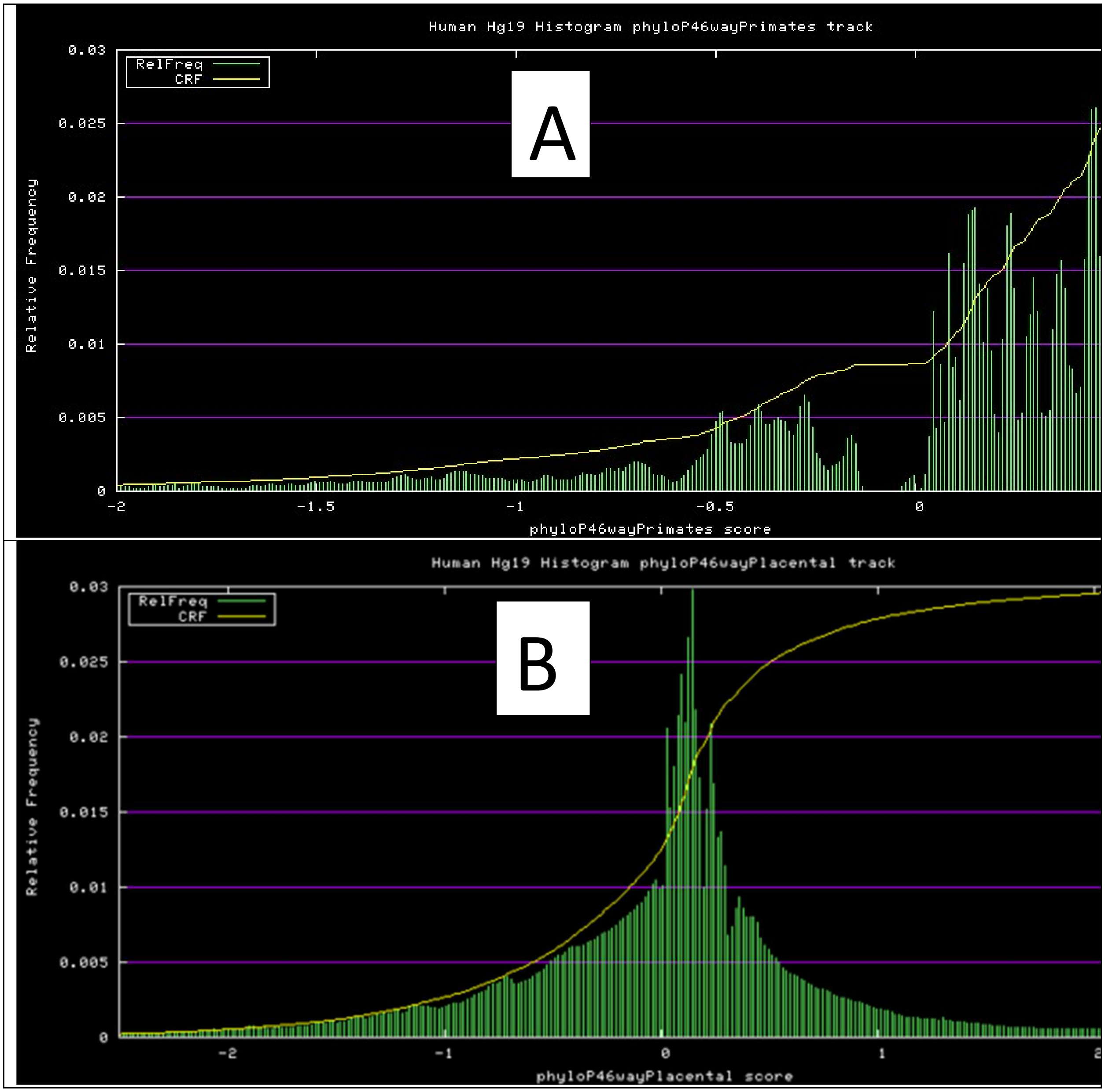
Human Hg19 Histogram phyloP46wayPrimates track
RelFreq
CRF
A
Relative Frequency
0.03
0.025
0.02
0.015
0.01
0.005
0
-2
-1.5
-1
-0.5
0
phyloP46wayPrimates score
Human Hg19 Histogram phyloP46wayPlacental track
RelFreq
CRF
B
Relative Frequency
0.03
0.025
0.02
0.015
0.01
0.005
0
-2
-1
0
1
2
phyloP46wayPlacental score

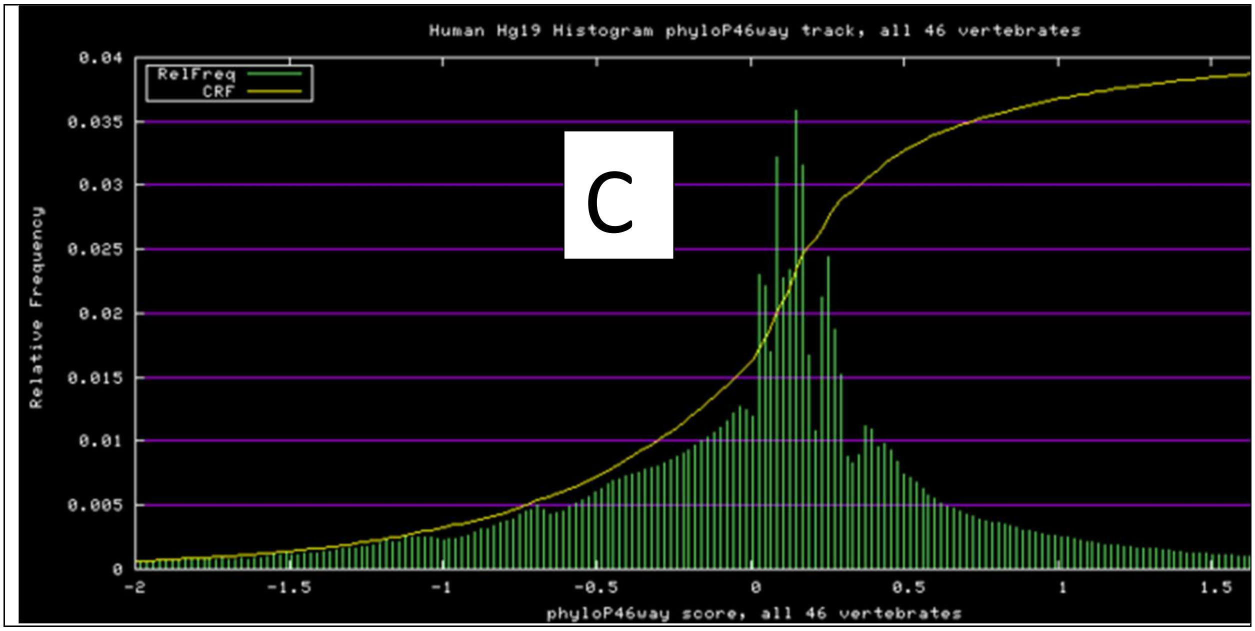

Figure S17. phyloP histogram analysis for mammal genomic tracks. Hg19 phyloP histograms (**https://genomewiki.ucsc.edu/index.php/Hg19_phyloP_histograms)** Histogram data for phyloP data on the 46-way conservation track on the human genome browser, hg19 each of these data tables have 2,845,303,719 data values**. (A**) Primate subset (primate subset data statistics: minimum: -9.065, maximum: 0.655, mean: 0.04482, standard deviation: 0.600051); (**B**) Placental mammal subset (minimum: -13.796, maximum: 2.941, mean: 0.03594, standard deviation: 0.779426), (**C**). All 46 vertebrates (minimum: -14.08, maximum: 6.424, mean: 0.0896, standard deviation: 0.833186).
